# Identification of Molecular Determinants in iRhoms1 and 2 That Contribute to the Substrate Selectivity of Stimulated ADAM17

**DOI:** 10.3390/ijms232112796

**Published:** 2022-10-24

**Authors:** Yi Zhao, Eliud Morales Dávila, Xue Li, Beiyu Tang, Ariana I. Rabinowitsch, Jose Manuel Perez-Aguilar, Carl P. Blobel

**Affiliations:** 1 Department of Biochemistry, Cellular and Molecular Biology, Weill Cornell Medicine, New York, NY 10021, USA; 2 School of Chemical Sciences, Meritorious Autonomous University of Puebla (BUAP), University City, Puebla 72570, Mexico; 3 Department of Pharmacology, Weill Cornell Medicine, New York, NY 10021, USA; 4 Weill Cornell/Rockefeller/Sloan-Kettering Tri-Institutional MD-PhD Program, New York, NY 10021, USA; 5 Department of Physiology, Biophysics and Systems Biology, Weill Cornell Medicine, New York, NY 10021, USA; 6 Arthritis and Tissue Degeneration Program, Hospital for Special Surgery, New York, NY 10021, USA

**Keywords:** ADAM17 (a disintegrin and metalloprotease 17), iRhom1 and 2 (inactive rhomboid like protein 1 and 2), EGFR-ligand (epidermal growth factor receptor ligand), protein ectodomain shedding

## Abstract

The metalloprotease ADAM17 is a key regulator of the TNFα, IL-6R and EGFR signaling pathways. The maturation and function of ADAM17 is controlled by the seven-membrane-spanning proteins iRhoms1 and 2. The functional properties of the ADAM17/iRhom1 and ADAM17/iRhom2 complexes differ, in that stimulated shedding of most ADAM17 substrates tested to date can be supported by iRhom2, whereas iRhom1 can only support stimulated shedding of very few ADAM17 substrates, such as TGFα. The first transmembrane domain (TMD1) of iRhom2 and the sole TMD of ADAM17 are important for the stimulated shedding of ADAM17 substrates by iRhom2. However, little is currently known about how the iRhoms interact with different substrates to control their stimulated shedding by ADAM17. To provide new insights into this topic, we tested how various chimeras between iRhom1 and iRhom2 affect the stimulated processing of the EGFR-ligands TGFα (iRhom1- or 2-dependent) and EREG (iRhom2-selective) by ADAM17. This uncovered an important role for the TMD7 of the iRhoms in determining their substrate selectivity. Computational methods utilized to characterize the iRhom1/2/substrate interactions suggest that the substrate selectivity is determined, at least in part, by a distinct accessibility of the substrate cleavage site to stimulated ADAM17. These studies not only provide new insights into why the substrate selectivity of stimulated iRhom2/ADAM17 differs from that of iRhom1/ADAM17, but also suggest new approaches for targeting the release of specific ADAM17 substrates.

## 1. Introduction

ADAM17 is a membrane-anchored metalloprotease that plays important roles in the regulation of the EGFR, TNFα, IL-6R, and other signaling pathways [1,2,3]. ADAM17 was initially discovered as the TNFα convertase, which is required for the release of the pro-inflammatory cytokine TNFα from the cell membranes [4,5]. Subsequent studies showed that the release of soluble TNFα from myeloid cells in vivo in the context of endotoxin shock and inflammatory arthritis depends primarily on ADAM17 [6,7]. ADAM17 is also essential for EGFR signaling since several EGFR-ligands, all of which are membrane anchored, must be released from the membrane by ADAM17 to become functionally active [8,9]. Mice lacking ADAM17 die shortly after birth with open eyes and defects in their heart valves and long bone growth plates. These pathological developmental phenotypes are caused by defects in EGFR activation and underscore the critical role of ADAM17 in EGFR signaling in vivo [6,10,11,12]. The vital contribution of ADAM17 to EGFR signaling also helps explain why patients with inactivating mutations in ADAM17 suffer from severe deficiencies in their skin and intestinal barrier [13,14,15], as the maintenance of these barriers depends on the cleavage of the ADAM17 substrate TGFα and subsequent EGFR signaling [16,17,18,19]. Conditional knockout mice and hypomorphic mice for ADAM17 have provided additional information about its role in different cells and tissues, which is often related to EGFR signaling [11,12,16,17,20]. Finally, ADAM17 has a crucial role in regulating IL-6/IL-6R signaling and is essential for the release of numerous other membrane proteins from cells [2,3,21].

The maturation and function of ADAM17 is controlled by the seven-membrane-spanning inactive rhomboid-like proteins iRhom1 and 2 (iR1, iR2) [22,23,24,25,26]. iR2 is the main regulator of ADAM17 in myeloid cells, where little, if any, iR1 is expressed [7]. In most other tissues, both iR1 and iR2 are present, except for the brain, where mainly iR1, but only low levels of iR2, are found [7,22]. Similar to conditional knockout mice lacking ADAM17 in myeloid cells, *iR2*−/− mice are healthy and viable, with no spontaneous pathological phenotypes, but are nevertheless protected from the pathogenic effects of soluble TNFα in mouse models for LPS-endotoxin shock and inflammatory arthritis [7,24]. Studies in cells that express both iR1 and iR2, such as mouse embryonic fibroblasts and chondrocytes, uncovered functional differences in the substrate selectivity of ADAM17 in wild-type cells versus *iR2*−/− cells where only iR1 was present [23,27]. iR2 can support stimulated shedding of the eight substrates of ADAM17 analyzed to date, whereas iR1 can only support stimulated shedding of three of these, including TGFα [23,27]. Specifically, inactivation of iR1 doesn’t affect the stimulated shedding of EGFR ligands or other membrane proteins by ADAM17, whereas the stimulated shedding of three out of five ADAM17-dependent EGFR ligands (AREG, HB-EGF and EREG) is reduced or abolished in *iR2*−/− cells. These observations suggest that iR1 and iR2 have a different modus of interacting with their substrates, thereby controlling the substrate selectivity of ADAM17 under stimulated conditions. Previous studies on the regulation of ADAM17 by iR2 have shown that the transmembrane domain (TMD) of ADAM17, and the TMD1 and cytoplasmic domain of iR2, are important for stimulation of ADAM17/iR2 [28,29]. Moreover, sequences in the cytoplasmic domain of iR2 have been implicated in the regulation and maturation of ADAM17 [30,31,32,33]. However, to our knowledge, little, if anything, is known about how the substrate selectivity of stimulated ADAM17 is controlled by iR1 and iR2.

To address this question, we generated chimeras between iR1 and iR2 to determine which parts of these multi-membrane-spanning proteins are responsible for the stimulated processing of the EGFR-ligands TGFα (iRhom1 or 2-dependent) and EREG (iRhom2-selective) [23]. Based on our results, we performed molecular dynamics simulations of iR1 and iR2 in complex with these two substrates to aid in understanding the difference in substrate selectivity of ADAM17/iR1 and ADAM17/iR2. Our results suggest that the processing of EREG by stimulated ADAM17/iR2 but not stimulated ADAM17/iR1 can potentially be explained by differences in how iR1 and iR2 present the cleavage site of EREG to the catalytic site of ADAM17.

## 2. Results

### 2.1. Chimeras between iRhom1 and iRhom2 Reveal a Key Contribution of the 7th Transmembrane Domain to the Substrate Selectivity of ADAM17

**Figure 1:**
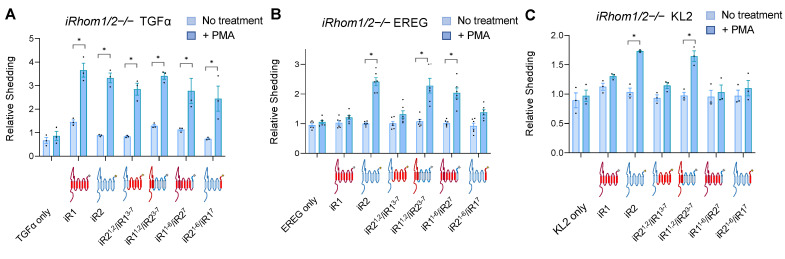
Characterization of the contribution of the TMDs of iR1 and iR2 to the substrate selectivity of stimulated ADAM17. Ectodomain shedding assays were performed in *iR1/2−/−* mEFs co-transfected with iR1 or iR2 or chimeras between iR1 and iR2 (iR1, iR2, iR2^1,2^/iR1^3–7^, iR1^1,2^/iR2^3–7^, iR1^1–6^/iR2^7^, iR2^1–6^/iR1^7^, see Supplementary Figure S1 for details) and TGFα (**A**), EREG (**B**) or KL2 (**C**). In the diagrams below each graph, components of iR1 are shown in red, and components of iR2 are shown in blue. Constitutive (no treatment) and PMA-stimulated shedding (+25 ng/mL PMA) for each variable were measured after 1 h of stimulation. Results are shown as mean ± SEM (*n* = 3 for (**A**,**C**), *n* = 6 for (**B**)), * indicates *p* ≤ 0.005 in an unpaired *t*-test between the untreated and PMA-treated condition for a given sample.

To explore the contribution of different domains of iRhom1 (iR1) or iRhom2 (iR2) to the substrate selectivity of ADAM17, we generated chimeras with the first two transmembrane domains (TMDs) of iR2 fused to the remaining five TMDs of iR1 (iR2^1,2^/iR1^3–7^) or vice versa (iR1^1,2^/iR2^3–7^). In addition, we constructed chimeras with only the last TMD of one iRhom attached to the first six TMDs of the other iRhom (iR2^1–6^/iR1^7^; iR1^1–6^/iR2^7^, see Supplementary Figure S1 for details). As shown in Figure 1A, when these chimeras were transfected into *iR1/2*−/− double knockout mEFs, all were able to restore the stimulation of TGFα shedding by the phorbol ester PMA (phorbol 12-myristate 13-acetate), which can be supported by either iR1 or iR2 [23]. A Western blot of the chimera probed with antibodies against their C-terminal T7 tag is shown in Supplementary Figure S2. The constitutive shedding of TGFα was lower in cells transfected with iR2, or with mutants carrying the TMD1 of iR2 compared to iR1, or mutants with the TMD1 of iR1 in shedding experiments performed for 1 h (Figure 1A). This observation was corroborated in shedding experiments over 3 h, which allow a better determination of differences in constitutive shedding (Supplementary Figure S3A). We also observed lower constitutive shedding of TGFα in *iR1*−/− mEFs, which only express iR2, than in *iR2*−/− mEFs, which only express iR1, or in wild-type mEFs, where both iR1 and iR2 are active, although the levels of PMA stimulated shedding were similar (Supplementary Figure S3B).

We next tested how these chimeras affected the stimulated shedding of the iR2-selective EGFR-ligand epiregulin (EREG), which was chosen here because it is the most iR2-selective EGFR-ligand [23,27]. We again stimulated cells with the phorbol ester PMA, which has a similar but stronger effect as physiological stimuli, such as GPCR agonists (LPA, Thrombin), or addition of PDGF or TNFα and is therefore often used as representative model stimulus for ADAM17 [23,34]. We found that iR1 and iR2^1,2^/iR1^3–7^ did not significantly increase constitutive or stimulated EREG shedding, whereas iR1^1,2^/iR2^3–7^ behaved more like wild-type iR2, in that it supported stimulated, but not constitutive, EREG shedding (Figure 1B). In addition, iR1^1–6^/iR2^7^ was able to rescue stimulated EREG shedding almost as well as iR2, whereas there was no significant increase by iR2^1–6^/iR1^7^. Finally, we tested how these chimeras affect the stimulated shedding of murine KitL2 (KL2), another highly iR2-selective substrate that has been used to study the regulation of iR2 and that is not an EGFR-ligand [23,28,29]. We found that stimulated KL2 shedding differed from stimulated EREG shedding, in that it was observed in the presence of iR1^1,2^/iR2^3–7^, whereas there was no significant increase in KL2 shedding in the presence of the other chimeras tested here, including iR1^1–6^/iR2^7^ that could stimulate EREG shedding (Figure 1C).

### 2.2. Specific Point Mutations Identify Amino Acid Residues in the TMD7 of iR2 That Are Required for Stimulated Shedding of EREG, but Not TGFα

**Figure 2:**
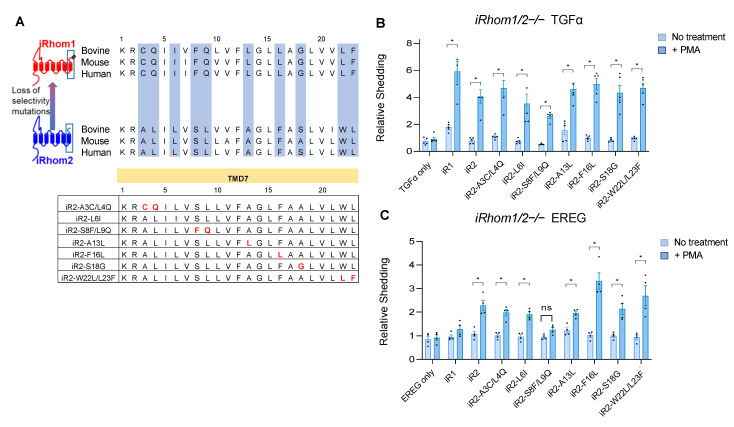

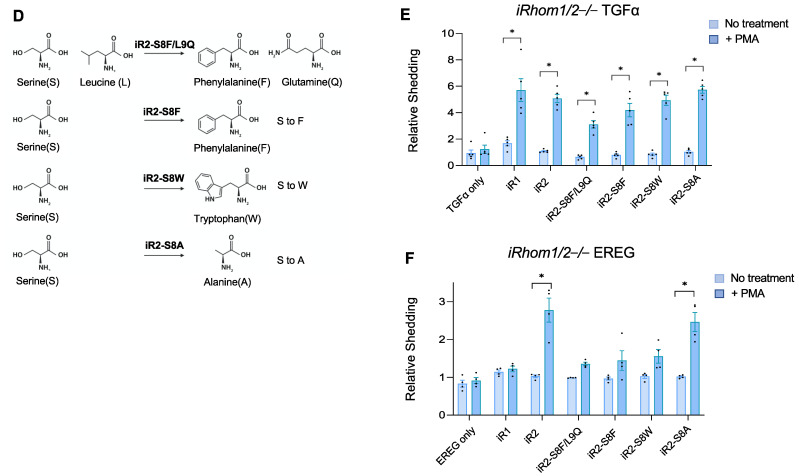
Point mutations in the TMD7 of iR2 identify key residues required for iRhom2-selective stimulated shedding of EREG. Point mutations in the TMD7 of iR2 were generated based on sequence alignments of the TMD7 in iR1 and iR2 from three species (human, mouse, bovine). (**A**) Amino acid residues that are highlighted in blue are different between iR1 and iR2 but are conserved between these three species for each iRhom. Point mutations in the TMD7 of iR2 to change the sequences highlighted in blue into the corresponding iR1 consensus sequence are shown in red letters in the lower panel. (**B**,**C**) Ectodomain shedding assays were performed in *iR1/2*−/− mEFs co-transfected with iR2 carrying the indicated point mutations (see panel (**A**) for details) and TGFα (**B**) or the iR2-selective EREG (**C**). (**D**) Chemical structures of the amino acid residues in position 8 and 9 of TMD7 of iR2 and of the amino acid residues that were used to replace these in the mutants tested in panels (**E**,**F**) (iR2-S808F/L809Q (labeled as iR2-S8F/L9Q); iR2-S808F (labeled as iR2-S8F); iR2-S808W (labeled as iR2-S8W) and iR2-S808A (labeled as iR2-S8A)). (**E**,**F**) Ectodomain shedding assays were performed in *iR1/2−/−* mEFs co-transfected with iR2 carrying the indicated point mutations in position 8 and 9 or only in position 9 (see panel (**D**)) together with TGFα (**E**) or EREG (**F**). Results are shown as mean ± SEM (*n* = 5 for (**B**), *n* = 4 for (**C**), *n* = 5 for (**E**), *n* = 4 for (**F**)), * indicates *p* ≤ 0.005 in a *t*-test between the untreated and PMA (+) condition for a given sample.

Since replacing the C-terminal TMD7 and extracellular C-terminal domain (ECTD) of iR2 with that of iR1 (iR2^1–6^/iR1^7^, see Figure 1) reduced the stimulated shedding of EREG to levels comparable with iR1, we tested whether point mutations in the TMD7 or ECTD of iR2 would affect the stimulated shedding of EREG compared to TGFα. For this purpose, we first aligned the amino acid sequences of the TMD7 and ECTD of human, mouse and bovine iR1 and iR2 to identify specific residues that are conserved between these three mammalian species in all three iR1 orthologs or iR2 orthologs, but differ between iR1 and iR2 (see Figure 2A for TMD7, Supplementary Figure S4A for the ECTD, relevant sequences highlighted in blue). Based on this alignment, we mutated specific amino acid residues in the iR2 TMD7 and ECTD consensus to those present in the corresponding positions in iR1. None of the mutants abolished the PMA-stimulated shedding of TGFα, although there was some variability in the level of PMA stimulation (Figure 2B, Supplementary Figure S4B). All iR2 mutants except for iR2-S808F/L809Q in the TMD7 (labeled as iR2-S8F/L9Q in Figure 2B,C) were able to support stimulated shedding of EREG, whereas iR2-F816L in TMD7 enhanced PMA-stimulated shedding of EREG compared to the wild-type control (Figure 2C, labeled as iR2-F16L, ECTD mutants shown in Supplementary Figure S4C). Further analysis showed that the iR2-S808F point mutation (iR2-S8F) as well as replacement of serine in position 808 with tryptophan, which also has a bulky side chain (iR2-S808W, shown as iR2-S8W in Figure 2D–F), significantly impaired the stimulated shedding of EREG, but not of TGFα compared to wild-type iR2 (Figure 2E,F). However, when the serine at position 808 in the iR2 TMD7 was replaced with alanine, which has a small side chain (iR2-S808A, labeled as iR2-S8A in Figure 2D–F), there was no significant effect on the stimulated shedding of EREG or TGFα compared to the wild-type iR2 control (Figure 2E,F). Taken together, these results demonstrate that a single point mutation in the TMD7 of iR2, replacing the serine in position 808 with an aromatic hydrophobic amino acid residue with a large side chain, significantly reduced the ability of iR2 to support stimulated shedding of EREG, but not of TGFα.

### 2.3. A Gain-of-Function Point Mutation in the TMD7 of iR1 Allows It to Promote a Modest but Significant Stimulation of Shedding of the iR2-Selective EREG

**Figure 3:**
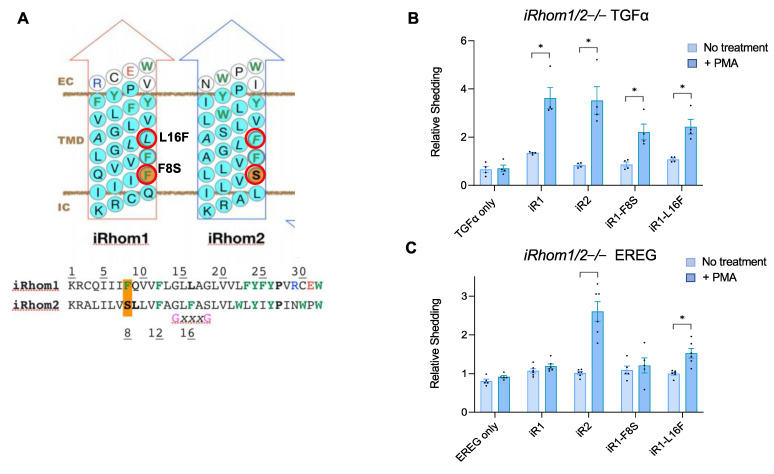
Point mutations in the TMD7 of iR1 allow it to support limited stimulation of EREG shedding. (**A**) Schematic representation of the TMD7 of iR1 and iR2 as an α-helix. Point mutations were designed to change amino acid residues on the same face of the TMD7 α−helix (red circles, iR1-F807S, labeled as iR1-F8S), iR1-L815F (labeled as iR1-L16F)). Amino acid residues with large side chains (W, Y, F) are shown in green letters. The position of iR2-S808 that is important for the selectivity of iR2 (see Figure 2F) and the corresponding iR1-F807 are marked in orange (**B**,**C**) Ectodomain shedding assays were performed in *iR1/2*−/− mEFs co-transfected with iR1 carrying the point mutations indicated in (**A**) and with TGFα (**B**) or EREG (**C**). Results are shown as mean ± SEM (*n* = 3), * denotes *p* ≤ 0.005 between the unstimulated and PMA (+) condition for a given sample.

Since replacing the TMD7 of iR1 with that of iR2 (iR1^1–6^/iR2^7^, Figure 1B) was sufficient to restore the stimulated shedding of EREG to levels comparable to those observed with WT iR2, we decided to replace specific amino acid residues in the TMD7 of iR1 with the corresponding amino acids found in iR2 to determine whether individual mutations would have a similar effect. Specifically, we focused on two residues situated on the same protein face of the α-helix of TMD7 as S808 in the loss-of-function iR2 mutants described above (Figure 3A, iR1-F807S, iR1-L815F, labeled iR1-F8S and iR1-L16F, respectively, to match the numbering of the corresponding iR2 residues in TMD7 shown in Figure 2). Both mutants could support the PMA-stimulated shedding of TGFα, although at somewhat reduced levels compared to WT iR1 (Figure 3B). When we analyzed stimulated EREG shedding, we found that the iR1-F807S mutant (iR1-F8S in Figure 3) behaved similarly to WT iR1, whereas the iR1-L815F (iR1-L16F in Figure 3) mutant supported a small, but significant increase in PMA-stimulated shedding of EREG compared to unstimulated cells (Figure 3C). Thus, even subtle changes in the putative interaction site of iR1 can differentially affect the efficiency of ADAM17-dependent processing of its substrates TGFα (reduced in both mutants) and EREG (enhanced by iR1-L815F (iR1-L16F in Figure 3).

### 2.4. Computational Methods Suggest That Different Substrate Conformations Determine the Substrate Selectivity of ADAM17/iR2 versus ADAM17/iR1

To shed light onto the potential determinants of the substrate selectivity of ADAM17 in the presence of iR1 or iR2, we utilized computational modeling methods. Due to the lack of experimental information regarding the tertiary structure of both proteins, we used the structure predicted by the AlphaFold program [35,36] (see Materials and Methods for details). The sequences and structures utilized are shown in Supplementary Figures S5–S7. The iR1 and iR2 structures were investigated by all-atom MD simulations using the program NAMD and the CHARMM36 force field [37,38]. The proteins were embedded in a hydrated phospholipid bilayer and simulated at a temperature of 37 °C. After 300 ns, the final structures of iR1 and iR2 were selected for further investigations regarding their interactions with the substrates EREG and TGFα.

Similar to the case of iR1 and iR2, the tertiary structure of the EREG and TGFα substrates were selected from the predictions of AlphaFold (substrate structure predictions shown in Supplementary Figure S7A,B, see Materials and Methods for details). In the case of EREG, the protein segment used comprised the EGF-like extracellular domain and the transmembrane domain for a total of 90 residues (see Supplementary Figure S7A). For TGFα, the segment utilized included the EGF-like extracellular domain and the transmembrane domain for a total of 91 residues (see Supplementary Figure S7B). To predict possible molecular poses for EREG in the structural framework of the iR1, docking studies were carried out using Autodock Vina [39] (see Section 4). Based on our experimental results, molecular poses that placed the TMD domain of EREG in proximity to the TMD7 of iR1 were favored (Supplementary Figure S8). Similar criteria were used for the other three protein complexes (see Supplementary Figure S9 for the molecular poses considered for TGFα and iR2, and Supplementary Figures S10 and S11 for the starting poses for all four complexes, including iR2/EREG and iR1/TGFα).

### 2.5. Unbiased Molecular Dynamics Simulations of the Protein Complexes

**Figure 4:**
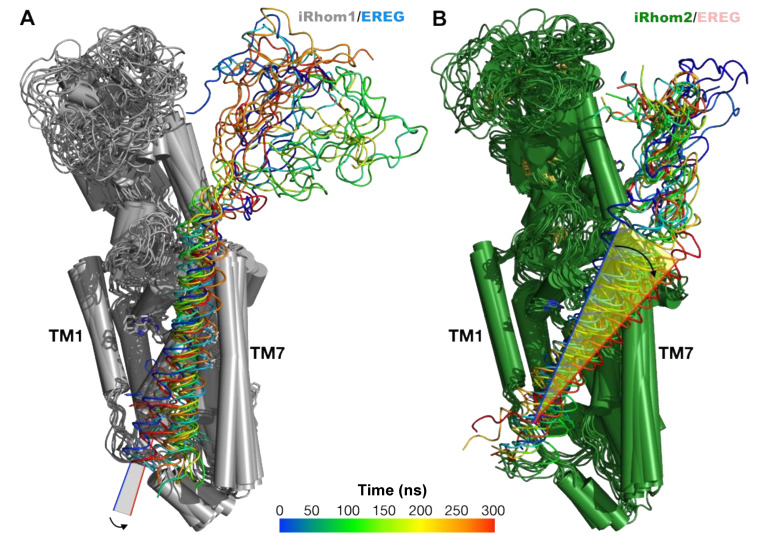
Molecular dynamics simulation of the iR1/EREG and iR2/EREG complexes. Superimposed structures of the iR1/EREG (**A**) and iR2/EREG (**B**) complexes at different times along the 300ns-long molecular dynamics simulation trajectories. The color code represents different times along the simulations for the EREG ligands, spanning from the initial complex structure (blue) toward the final structure of the simulations (red); see also color bar. The structure of iR1 is colored gray while that for iR2 is colored green. A change in the tilt of the TMD domain of the substrate in the iR2/EREG complex, relative to the position of the TMD7 of iR2, is highlighted with an arrow.

**Figure 5:**
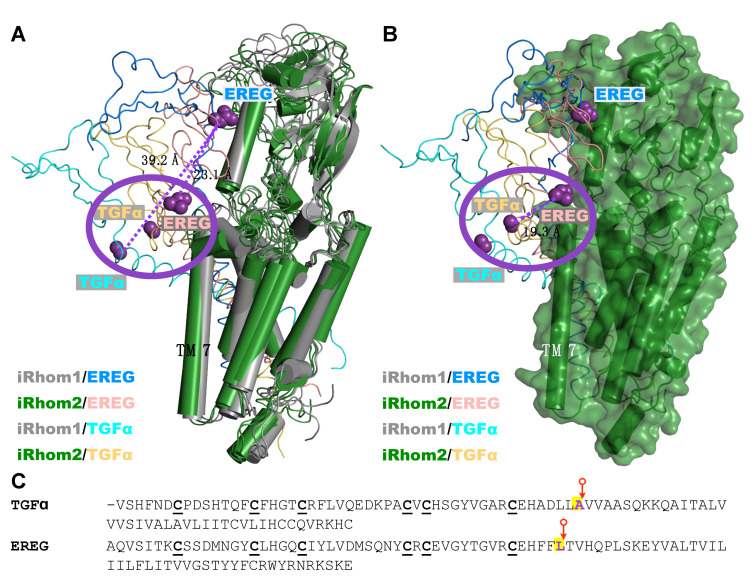
Representative structure of the final stages of the four iRhom/substrate complexes. (**A**) Overlapping structures of the four protein complexes, iR1/EREG, iR2/EREG, iR1/TGFα, and iR2/TGFα, are shown. Residue positions flanking the distal extracellular cleavage sites of TGFα and EREG are shown in purple. A purple oval is presented to highlight the proximity of this position in the iR2/EREG, iR1/TGFα and iR2/TGFα complexes but not in the iR1/EREG system. (**B**) Similar representation of the four ligand structures but the iR1 structure is excluded for clarity. In this surface representation, the difference in the purple residue is more evident and accentuates the difference in positioning, and thus presumably accessibility to this segment. When the relative position between the substrate cleavage sites (colored purple) is compared for complexes of iR2 with TGFα (iR1 or 2-dependent) and EREG (iR2-selective), the distance between the Cα atoms is 19.3 Å (see dashed line in panel (**B**)). However, in the case of the complexes with iR1, the distance between the equivalent substrate cleavage sites of TGFα and EREG is more than double, that is, 39.2 Å (dashed line in panel (**A**)). Additionally, the distance between the substrate cleavage sites in the EREG substrate in complex with iRhom2 versus iRhom1 is significant (23.1 Å, see dashed line). (**C**) The amino acid residue sequence of the two ligands is shown, beginning at the EGF-repeat with six conserved cysteine residues (bold and underlined) and ending at the cytoplasmic side of the TMD. The location of the membrane-proximal extracellular cleavage site for ADAM17 in TGFα and EREG is indicated by a red arrow.

Four protein complexes were investigated by unbiased MD simulations, iR1/EREG, iR2/EREG, iR1/TGFα, and iR2/TGFα (see Supplementary Figure S12 for a representative set up of the simulated system). Figure 4 shows superimposed structures of the iR1/EREG and iR2/EREG complexes at different times along the 300ns-long trajectories. Our results indicate that there is a difference in the tilt of the helical TMD domain of EREG, relative to the position of the iR1 (in gray) and iR2 (green) structure, particularly with the TMD7 helix (Figure 4A,B, see also Supplementary Figure S13). This difference may be attributed, at least in part, to the presence of aromatic residues in the TMD domain of EREG (Supplementary Figure S14B, such as residue F129 in EREG) and also in the different positions along the TMD7 of iR1/2, including residue F816 in iR2 (L815 in iR1, see Supplementary Figures S11F and S14A). In contrast, the results for the iR1/TGFα and iR2/TGFα complexes indicated that the conformations adopted by the substrate TMD domains were similar in both complexes. Interestingly, when the position of the two substrates in all the complexes are superimposed, there are similarities between the iR2/EREG, iR1/TGFα, and iR2/TGFα complexes, but not with iR1/EREG (see Figure 5A,B), as indicated by the position of the extracellular cleavage sites for ADAM17 in TGFα and EREG [40] (pointed to by a red arrow in Figure 5C, indicated by purple spheres in Figure 5A,B). An overlay of iR1 TMD7 with iR2 TMD7 shows substantial differences in the predicted position of iR2-808S compared to the corresponding iR1-807F residue (Supplementary Figure S15). When we modeled EREG with the iR2-S808F point mutant, which strongly reduced EREG shedding in cell-based assays (see above, Figure 2F), and compared the outcome to wild-type iR2/EREG, we found a change in the tilt of the iR2-S808F/EREG TMD compared to wild-type iR2/EREG (Supplementary Figure S16). This resulted in a substantial displacement of the EREG cleavage site away from the position found in wild-type iR2 about half-way towards the position predicted for the iR1/EREG complex (Supplementary Figure S17). The different position of the cleavage site in iR1/EREG and iR2-S808F/EREG versus iR2/EREG, iR1/TGFα and iR2/TGFα provides a plausible explanation for the lack of EREG substrate cleavage by stimulated ADAM17/iR1 or ADAM17/iR2-S808F, in that it could conceivably make the cleavage site in EREG less accessible to the catalytic site of ADAM17.

## 3. Discussion

The main goal of this study was to explore how the iRhoms determine the substrate selectivity of ADAM17. The premise was the observation that ADAM17/iR2 can increase the processing of several ADAM17 substrates under stimulated conditions, such as following treatment with TNFα, the GPCR agonists LPA or thrombin, the growth factor PDGF, or the phorbol ester PMA, whereas ADAM17/iR1 can only promote the stimulated processing of very few substrates, such as TGFα [23]. The overall conservation of the domain organization of iR1 and 2, including the position of their seven transmembrane domains, allowed us to generate iR1/2 chimeras to identify which TMD or other region in iR2 was necessary to support stimulated shedding of the iR2-selective substrates EREG and KL2. In all experiments, the stimulated shedding of TGFα served as a positive control for the ability of the chimeras to support the function of ADAM17 and its ability to respond to stimulation by PMA [23,28,29]. The domain swaps identified the TMD7 of iR2 as an important determinant of the substrate selectivity of stimulated ADAM17/iR2. This conclusion was further supported by the lack of significant stimulated EREG-shedding by forms of iR2 with point mutations in their TMD7 that increased the size of the side chain of specific residues located on one side of the helical face. Moreover, we found that a point mutation on the same side of the helical face of iR1 allowed it to support some stimulated shedding of EREG. Our results highlight the importance of specific amino acid residues (808 and 816; *i* and *i* + 4 in a helical segment), which are located at the same protein interface in the α−helix of TMD7 of iR1 and iR2, for the substrate selectivity of stimulated ADAM17/iR2.

Since iR1 in the *iR2*−/− mEFs does not support the stimulated shedding of the iR2-selective EREG, yet can nevertheless activate TGFα shedding upon stimulation of ADAM17 with PMA, the ability of stimulated ADAM17/iR1 to gain access to the EREG cleavage site must differ from that of stimulated ADAM17/iR2. Previous studies have shown that PMA stimulation increases the turnover of a soluble TNFα peptide in the supernatant of the stimulated cells, which provided the first evidence that the activity of ADAM17 can be post-translationally regulated on the cell surface [41,42]. Moreover, studies with the hydroxamate DPC333, a tight-binding inhibitor of ADAM17, have suggested that stimulation of ADAM17 allows DPC333 to gain access to its catalytic site, whereas unstimulated constitutively active ADAM17 molecules are much less accessible to DPC333 [34]. This suggested that the activation of ADAM17 by stimuli such as PMA correlates with a conformational change that allows DPC333 to gain access to its catalytic site. As the related ADAM10 has been shown to have an open and closed configuration of its catalytic site [43], this has led to a model in which the opening and closing of ADAM17 upon PMA stimulation is regulated through an allosteric switch that controls access to its catalytic site [29]. Moreover, studies with the hypomorphic iR2 sinecure mutant, which has a point mutation in the first TMD of iR2, and with ADAM17 mutants carrying point mutations in its transmembrane domain suggest that the allosteric switch that exposes the catalytic site of ADAM17 can be triggered by iR2 [28,29]. Since both iRhoms can support stimulated shedding of TGFα, this suggests that both iR1 and iR2 should be able to activate the putative allosteric switch that opens and closes the catalytic site of ADAM17 [29]. However, the inability of activated ADAM17/iR1 to enhance the shedding of several substrates of ADAM17, such as EREG and KL2, suggests that the catalytic site of ADAM17/iR1 does not have access to these substrates upon stimulation, whereas the activated ADAM17/iR2 does [23,28,29]. Taken together, these observations raised the possibility that the substrate selectivity of the stimulated form of ADAM17 is regulated, at least in part, through differences in the presentation and accessibility of the substrate cleavage site to the active and open catalytic site of stimulated ADAM17/iR1 versus ADAM17/iR2.

To further explore this possibility, we turned to computational methods to evaluate the interaction between iR1 and iR2 with the non-selective substrate TGFα and the iR2-selective EREG. In a recently published structural model of iR1 and iR2 (AlphaFold), the TMD7, which regulates the substrate selectivity of both iRhoms, is found close to TMD1, which is where previous studies have placed the interaction with the single TMD of ADAM17 [28,29] (see also Supplementary Figure S18). Binding of the substrates to the TMD7 of iR1 or iR2 could thus help bring their cleavage site close to the catalytic site of ADAM17. The observation that all iRhom mutants tested here supported the stimulated shedding of TGFα suggests that the proposed conformational change that opens up the catalytic site of stimulated ADAM17 [29,43] is not affected by these mutants. Instead, our results support a model in which the substrate selectivity ADAM17/iR2 versus ADAM17/iR1 depends on how exactly the TMD7 of each iRhom interacts with and presents the substrate cleavage site to ADAM17. This is most likely accomplished in coordination with additional interactions in the adjacent juxtamembrane and extracellular domains of the substrates and the iRhoms.

Consistent with this notion, we found that structural modeling predicted different poses and orientations for the interactions of iR1 and iR2 with TGFα versus EREG. Specifically, the cleavage site of iR1/TGFα, iR2/TGFα and iR2/EREG were in close proximity to one another, whereas the cleavage site of iR1/EREG was quite removed from the position of the other three cleavage sites (see Figure 5). If we use the location of the cleavage sites of iR1/TGFα, iR2/TGFα and iR2/EREG to operationally define positions that can be reached by the catalytic site of stimulated ADAM17, this could explain how iR2 might be able to present the cleavage site of ADAM17 substrates differently from iR1. This interpretation is further supported by modeling of the complex between EREG and the iR2-S808F point mutation, which strongly reduced stimulated EREG shedding in functional assays. The predicted iR2-S808F/EREG complex had a markedly different orientation of the EREG TMD and a different position of the EREG cleavage site compared to iR2/EREG. The resulting displacement of the EREG cleavage site in the iR2-S808F/EREG complex compared to the iR2/EREG complex could conceivably prevent access of the catalytic site of stimulated ADAM17, providing a plausible explanation for how this mutation affected the processing of EREG. In addition, other interactions that are not modeled here might have a role in determining the substrate selectivity of ADAM17/iR1 versus ADAM17/iR2, such as in the extracellular or cytoplasmic domains of the iRhoms and their substrates. Further studies, including additional structure/function analysis of the iRhoms and their different substrates, as well as structural studies of complexes between ADAM17/iRhoms and substrates, such as by Cryo-EM, will be necessary to further corroborate these models. Finally, it should be noted that a recent study identified a different means of regulating the substrate selectivity of ADAM17 towards TNFα that is based on an interaction of TNFα with Tspan8 and ADAM17 and recruitment of the enzyme and substrate in Tspan-enriched microdomains [44].

Given the known role of the EGFR in pathologies such as cancer and auto-immune diseases [45,46,47], the physiological and translational significance of our findings is that they provide new insights into how the processing and functional activation of EGFR-ligands by ADAM17 may be regulated. We propose that iR2 can position iR2-selective substrates such as EREG in a way that their cleavage site is accessible to the catalytic site of stimulated ADAM17, whereas iRhom1 might not be able to properly align stimulated ADAM17 with the cleavage site of EREG to allow its processing. Further studies will be necessary to determine whether a similar mechanism controls the substrate selectivity of ADAM17/iR2 towards other substrates. A better understanding of this process might allow a selective inactivation of the shedding of one or more EGFR-ligands or other selective substrates, such as KL2 [23], by targeting the interaction of iR2 with its selective substrates. Our results suggest that it might be possible to screen for molecules that selectively affect the orientation or presentation of the cleavage site(s) of EGFR ligands and other substrate proteins to ADAM17/iR2. This could help block the activity of EGFR ligands implicated in cancer and inflammation (e.g., AREG, EREG, [48,49,50,51,52]) without affecting the function of TGFα in protection of the skin and intestinal barrier [13,16,17]. This, in turn, could provide novel treatment options for diseases that depend on the dysregulated processing of specific EGFR ligands.

## 4. Materials and Methods

### 4.1. Cell Lines and Reagents

All materials were from Sigma-Aldrich (St. Louis, MO, USA) unless mentioned otherwise. The QuikChange II XL Site-Directed Mutagenesis Kit was from Agilent (Santa Clara, CA, USA, catalog #200521). Restriction enzymes and molecular biology reagents were from New England BioLabs (Ipswich, MA, USA, Xbal, #R0145S; Xhol, #R0146S; T4 DNA ligase, #M0202S; Stable Competent *E. coli* (High Efficiency), #C3040H). Anti-T7-tag (D9E1X) rabbit mAB were from Cell Signaling Technology (Danvers, MA, USA); anti-GAPDH mouse mAB from ABclonal (Woburn, MA, USA) and anti-rabbit and anti-mouse HRP-tagged secondary antibodies were from Promega (Madison, WI, USA). Mouse embryonic fibroblasts (mEFs) that had been isolated from embryonic day (E) 13.5 *iR1/2−/−* embryos have been described previously [22,23].

### 4.2. Cloning and Generation of iRhom1/2 and Substrate Mutants

The expression vectors for alkaline phosphatase (AP)-tagged EGFR ligands (TGFα, EREG) and KitL2, as well as the vectors for murine iR1 and iR2, have been described previously [8,53]. The chimeras between iR1 and iR2 were generated by overlap extension PCR using pcDNA 3.1(+)-iR1-T7 and pcDNA 3.1(+)-iR2-T7 as a template. The chimeras were cloned into a pcDNA 3.1(+) backbone and sequenced. Point mutations in iR1 and iR2 were generated using the QuikChange II XL Site-Directed Mutagenesis Kit, following the manufacturer’s instructions. The primers for the point mutations are listed in Table 1, with the mutations shown in red.

### 4.3. Ectodomain Shedding Assays

mEFs of the indicated genotypes were seeded on 12-well tissue culture plates to reach 80% confluency the next day. The cells were washed and starved with OptiMEM (OPTI-Eagle’s minimal essential medium, Thermo-Fisher, Waltham, MA) and transfected with AP-tagged TGF or AP-tagged KitL-2 (KL2) with or without iR1 or iR2 or mutant iR1 or iR2 constructs, using lipofectamine 3000 (Thermo-Fisher, Waltham, MA, USA). The transfection reagents were removed after 5 h incubation and replaced with Dulbecco’s Modified Eagle Medium (DMEM) with 10% fetal bovine serum (Atlanta Biologicals, Flowery Branch, GA, USA) and 1% penicillin/streptomycin. The next day, when the cells were 100% confluent, they were starved in OptiMEM for 1 h, followed by stimulation with or without PMA (phorbol 12-myristate 13-acetate) at 25 ng/mL for 1 h. The cell lysates and supernatants were harvested and incubated with the AP substrate 4-nitrophenyl phosphate and the absorbance at OD405nm was measured after incubation at room temperature overnight. The supernatant of a well that was not transfected was used as negative control. The levels of constitutive shedding and stimulated shedding were measured by determining the absorbance at OD405nm for each sample, and the percentage of shedding was calculated as the ratio of the absorbance at OD405nm in the supernatant over the absorbance of lysate plus supernatant, after subtracting the values of the untransfected supernatant and lysate control [54]. For normalization of the shedding experiments, the average of constitutive shedding results for all samples in the same experiments were calculated and set as 1, and the shedding levels in the same experiment were normalized to this average constitutive shedding and shown as a percentage. Each sample was measured in 3 identical wells, and each experiment was repeated at least 3 times.

### 4.4. Western Blot Analysis

Cells were transfected with the plasmids for iR1, iR2 or the chimera described above. The next day, the cells were lysed on ice in a cell lysis buffer (1% Triton in PBS, 10 mM phenanthroline, 1:250 protease inhibitor cocktail) and centrifuged at 13,000 rpm for 15 min at 4 °C to remove cell debris and nuclei. Then reducing agent (DTT) and 6x SDS sample buffer were added to the samples, which were incubated at 37 °C for 30 min. The samples were loaded at comparable amounts onto 10% SDS/polyacrylamide gels, separated for 30 min at 80 mV, then 90 min at 120 mV and transferred onto nitrocellulose transfer membranes. The membranes were then incubated on a shaker in blocking buffer (3% non-fat dry milk reconstituted in 0.05% Tween-20 in PBS) for 1 h, then incubated with an anti-T7 primary monoclonal antibody at 4 °C overnight. The next day, the membranes were washed and treated with secondary anti-T7 antibody. Then the membranes were washed 3 times in 0.05% Tween-20 in PBS, and bound antibodies were detected following incubation with ECL buffers (Amersham Biosciences, Amersham, UK) and imaged with a Chemdoc image analyzer (Bio-Rad, Hercules, CA, USA).

### 4.5. Computational Modeling

The tertiary structures of human iR1 and iR2 as well as the two substrates, EREG and TGFα, were obtained from the structures predicted by the AlphaFold program [35,36]. The accession codes for the AlphaFold structures are as follows: human iR1, Q96CC6; human iR2, Q6PJF5; EREG, O14944; and TGFα, P01135 (Supplementary Figures S5, S6 and S7A,B). iR1 was investigated by all-atom MD simulations. The systems were embedded in a hydrated phospholipid (POPC) bilayer and simulated at a temperature of 37 °C and 1 atmosphere (atm) of pressure, as previously described for other transmembrane proteins [55,56,57,58].

The system, comprising ∼90,000 atoms, was investigated by unbiased microsecond-length MD simulations using the program NAMD2.12 [37] and the CHARMM36 force field with CMAP corrections [38]. After 300 ns, the final structures of iR1 and iR2 were selected for further investigations regarding the interaction between iR1 and 1R2 and the EREG and TGFα substrates (see Figure S14).

The prediction of possible molecular poses for EREG ligand in the structural framework of iR1, were performed via docking studies using Autodock Vina [39]. Due to the complexity of the EREG ligand segment used (EGF-like extracellular and transmembrane domains for a total of 90 residues), its rotational bonds were constrained during the docking calculation, such that it was treated as a rigid body. From the experimental results, protein complexes that positioned the TMD domain of the substrate in proximity to the TMD7 of iR1 were favored. Additionally, energetic considerations (i.e., Autodock Vina scoring function) were used to select a representative structure of the iR1/EREG complex for further investigation using unbiased MD simulations. In the case of the iR2/EREG complex, a similar approach was used, and a similar protocol was followed for TGFα and iR1 and iR2. The initial structures are shown in Supplementary Figure S11.

## 5. Statistics

All values are expressed as mean ± standard error of the mean (SEM), using data from at least three independent experiments. The one-tailed Student’s *t*-test was used for all statistical analyses. *p* < 0.005 was considered statistically significant.

## Figures and Tables

**Table 1:** Primers for mutagenesis.

Name	Sequence	Direction
iR2-A3C/L4Q	5′-GTACCGCAAGCGATGCCAGATCCTCGTGTCG-3′	Forward
	5′-CGACACGAGGATCTGGCATCGCTTGCGGTAC-3′	Reverse
iR2-L6I	5′-CGAGCCCTCATCATCGTGTCGCTGC-3′	Forward
	5′-GCAGCGACACGATGATGAGGGCTCG-3′	Reverse
iR2-S8F/L9Q	5′-CCTCATCCTCGTGTTCCAGCTGGTCTTTG-3′	Forward
	5′-CAAAGACCAGCTGGAACACGAGGATGAGG-3′	Reverse
iR2-A13L	5′-CTGCTGGTCTTTCTTGGGCTCTTTGC-3′	Forward
	5′-GCAAAGAGCCCAAGAAAGACCAGCAG-3′	Reverse
Ir2-F16L	5′-GCTGGGCTCTTAGCTTCCCTGGTG-3′	Forward
	5′-CACCAGGGAAGCTAAGAGCCCAGC-3′	Reverse
iR2-S18G	5′-GCTCTTTGCTGGCCTGGTGCTGTGG-3′	Forward
	5′-CCACAGCACCAGGCCAGCAAAGAGC-3′	Reverse
iR2-W22L/L23F	5′-CCTGGTGCTGCTGTTCTACATCTACC-3′	Forward
	5′-GGTAGATGTAGAACAGCAGCACCAGG-3′	Reverse
iR1-F8S	5′-CAGATCATCATCTCCCAGGTCGTCTTCC-3′	Forward
	5′-GGAAGACGACCTGGGAGATGATGATCTG-3′	Reverse
iR1-L16F	5′-CTGGGCCTGTTTGCCGGCCTGGTG-3′	Forward
	5′-CACCAGGCCGGCAAACAGGCCCAG-3′	Reverse

## Supplementary Materials

The following supporting information can be downloaded at: https://www.mdpi.com/article/10.3390/ijms232112796/s1.
