# Supplementary material for: Identification of Molecular Determinants in iRhoms1 and 2 That Contribute to the Substrate Selectivity of Stimulated ADAM17
Source: Int J Mol Sci. 2022 Oct 24;23(21):12796. doi: 10.3390/ijms232112796 (PMC9654401; doi:10.3390/ijms232112796)
Supplement: Supplementary file 1 [file ijms-23-12796-s001.zip › ijms-1932283-supplementary.pdf]

Supplementary Figures for the manuscript entitled
Identification of molecular determinants in iRhoms1 and 2 that contribute to the substrate selectivity of stimulated ADAM17, by Zhao, Yi et al.

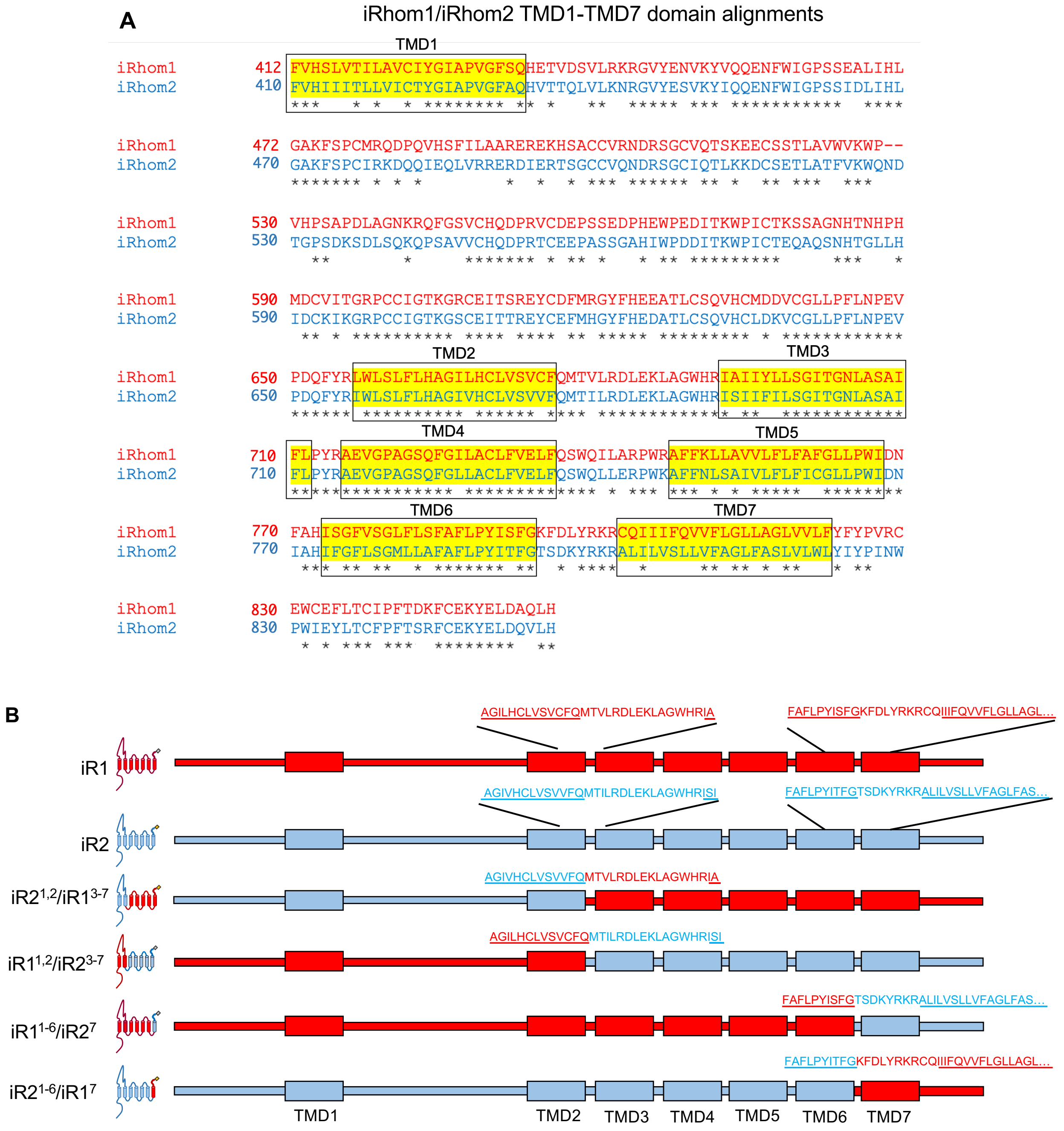

Supplementary Figure S1. Sequence alignment of iR1 and iR2 starting at the TMD1 of both proteins, with conserved amino acid residues indicated by an asterisk, and the TMDs highlighted in yellow and boxed (**A**). A diagram of the domain swap mutants is shown in (**B**).

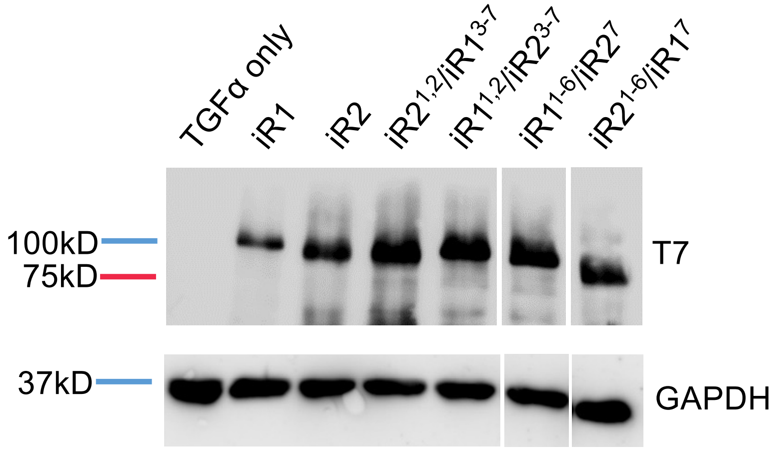

Supplementary Figure S2. Western blot analysis of the expression of the chimeric constructs shown in Supplementary Figure S1B in *iR1/2−/−* mEFs. All iRhom constructs carried a C-terminal T7 tag, so the Western blot was probed with anti-T7 (top panel) or with anti-GAPDH as loading control (lower panel).

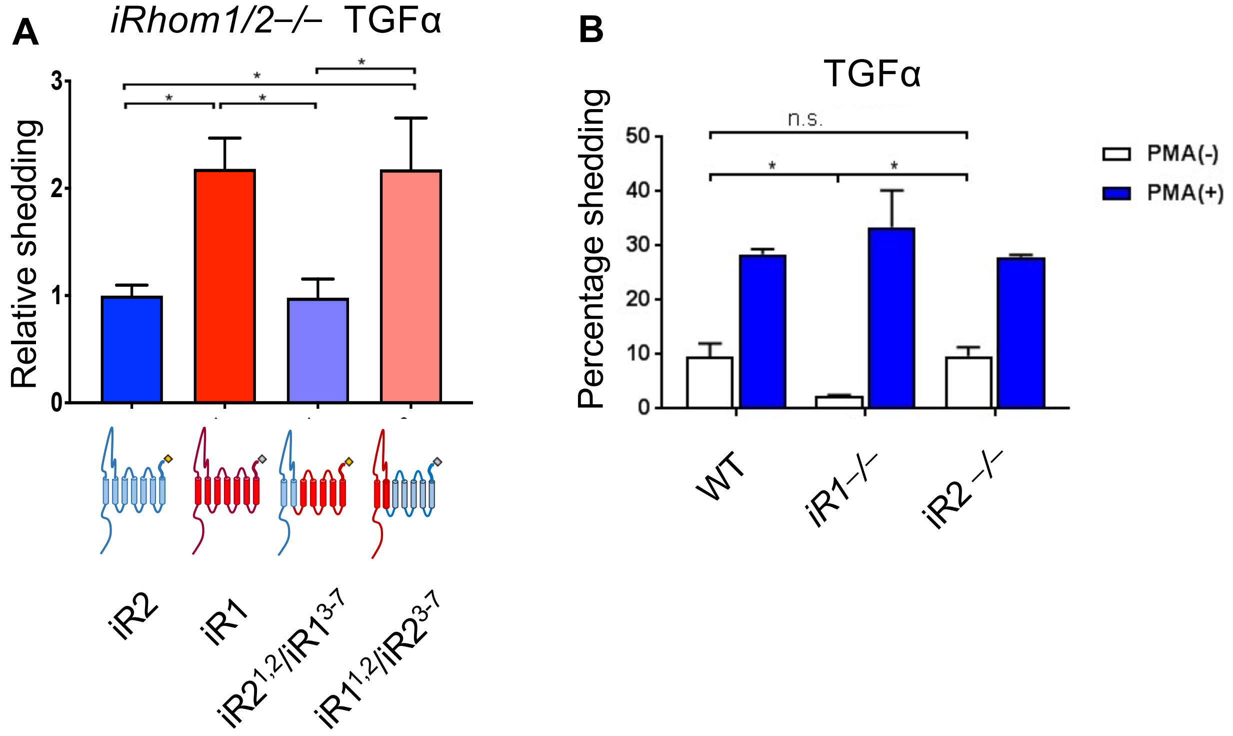

Supplementary Figure S3. Effect of overexpressed iR1 and iR2 in *iR1/2−/−* mEFs on constitutive shedding of TGFα into the supernatant over 3 hrs (**A**). Shedding of TGFα by endogenous iR2 (present in *iR1−/−* mEFs) or endogenous iR1 (present in *iR2−/−* mEFs) with or without stimulation with PMA over 1 hour (**B**).

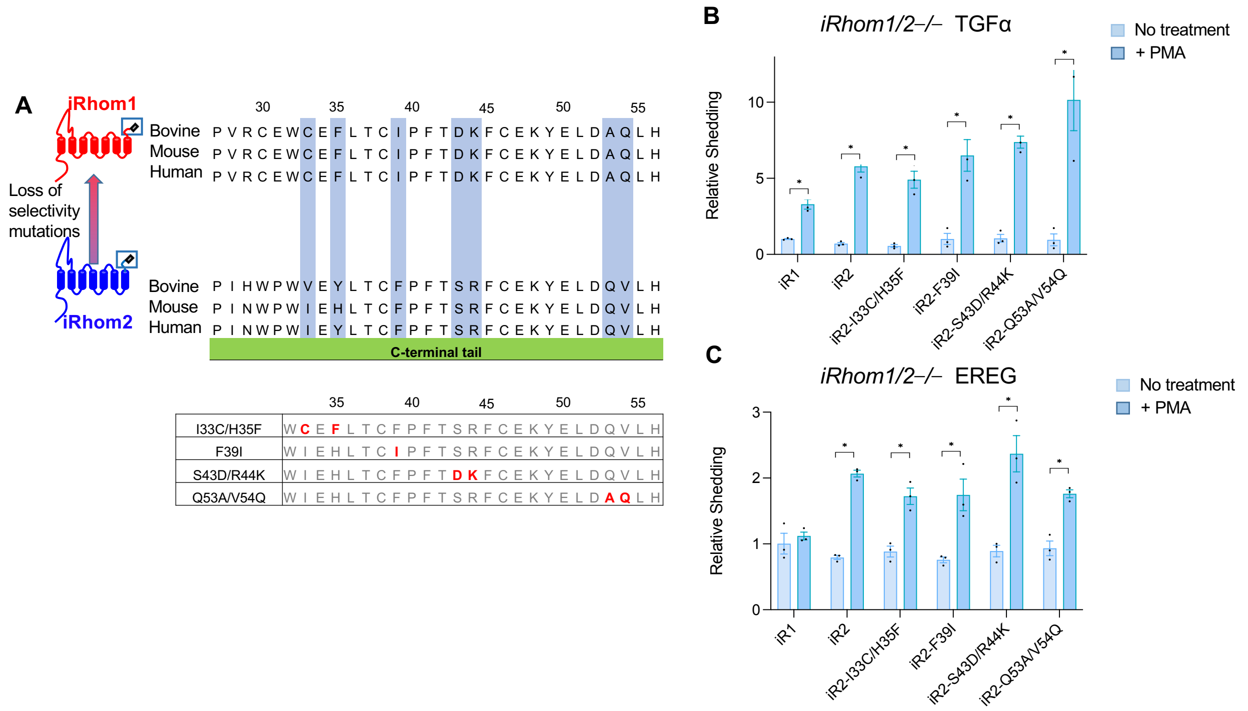

Supplementary Figure S4. Effect of point mutations in the extracellular C-terminal domain (ECTD) of iR2. (**A**) Amino acid residues labeled in blue in the ECTD differ in iR1 and iR2 but are conserved between human, mouse and bovine sequences for each iRhom. We introduced point mutations in the ECTD of iR2 to change the residues highlighted in blue to the corresponding iR1 consensus sequence, as shown in the lower panel. (**B, C**) Cell-based shedding assays in *iR1/2−/−* mEFs co-transfected with iR2 with the indicated point mutations (see panel **A** for details) and TGFα (**B**) or the iR2-selective EREG (**C**). Results are shown as mean ± SEM; *n* = 3, * indicates P ≤ 0.005 in a t-test between the untreated and PMA (+) condition for a given sample.

A UniProtKB - Q96CC6 (RHDF1_HUMAN)

```
        10         20         30         40         50
MSEARRDSTS SLQRKKPPWL KLDIPSAVPL TAEEPSFLQP LRRQAFLRSV
        60         70         80         90        100
SMPAETAHIS SPHHELRRPV LQRQTSITQT IRRGTADWFG VSKDSDSTQK
       110        120        130        140        150
WQRKSIRHCS QRYGKLKPQV LRELDLPSQD NVSLTSTETP PPLYVGPCQL
       160        170        180        190        200
GMQKIIDPLA RGRAFRVADD TAEGLSAPHT PVTPGAASLC SFSSSRSGFH
       210        220        230        240        250
RLPRRRKRES VAKMSFRAAA ALMKGRSVRD GTFRRAQRRS FTPASFLEED
       260        270        280        290        300
TTDFPDELDT SFFAREGILH EELSTYPDEV FESPSEAALK DWEKAPEQAD
       310        320        330        340        350
LTGGALDRSE LERSHLMLPL ERGWRKQKEG AAAPQPKVRL RQEVVSTAGP
       360        370        380        390        400
RRGQRIAVPV RKLFAREKRP YGLGMVGRLT NRTYRKRIDS FVKRQIEDMD
       410        420        430        440        450
DHRPFFTYWL TFVHSLVTIL AVCIYGIAPV GFSQHETVDS VLRNRGVYEN
       460        470        480        490        500
VKYVQQENFW IGPSSEALIH LGAKFSPCMR QDPQVHSFIR SAREREKHSA
       510        520        530        540        550
CCVRNDRSGC VQTSEEECSS TLAVWVKWPI HPSAPELAGH KRQFGSVCHQ
       560        570        580        590        600
DPRVCDEPSS EDPHEWPEDI TKWPICTKNS AGNHTNHPHM DCVITGRPCC
       610        620        630        640        650
IGTKGRCEIT SREYCDFMRG YFHEEATLCS QVHCMDDVCG LLPFLNPEVP
       660        670        680        690        700
DQFYRLWLSL FLHAGILHCL VSICFQMTVL RDLEKLAGWH RIAIIYLLSG
       710        720        730        740        750
VTGNLASAIF LPYRAEVGPA GSQFGILACL FVELFQSWQI LARPWRAFFK
       760        770        780        790        800
LLAVVLFLFT FGLLPWIDNF AHISGFISGL FLSFAFLPYI SFGKFDLYRK
       810        820        830        840        850
RCQIIIFQVV FLGLLAGLVV LFYVYPVRCE WCEFLTCIPF TDKFCEKYEL

DAQLH
```

B

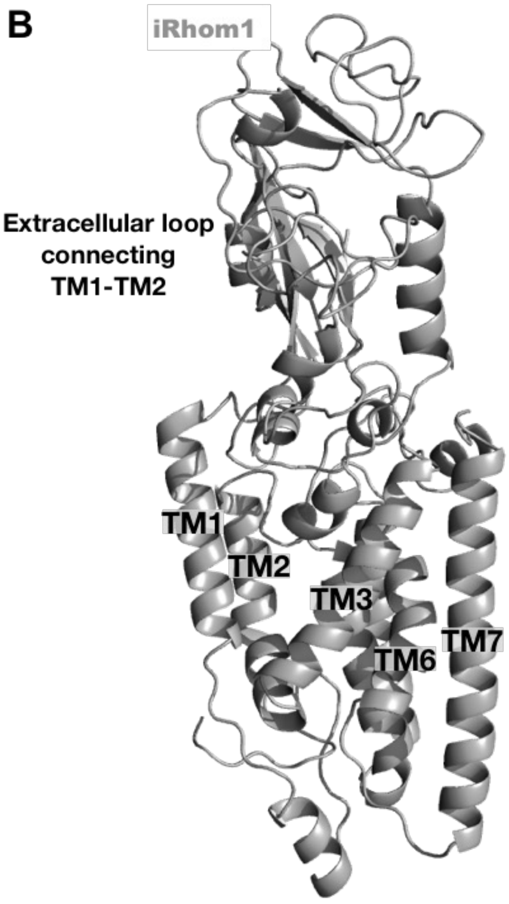

Supplementary Figure S5. Sequence (**A**) and predicted structure (**B**) of the human iR1 protein utilized in this study. (**A**) The cytoplasmic part of the protein sequence excluded in the 3D structure is shown in gray.

A UniProtKB - Q6PJF5 (RHDF2_HUMAN)

```
        10         20         30         40         50
MASADKNGGS VSSVSSSRLQ SRKPPNLSIT IPPPEKETQA PGEQDSMLPE
        60         70         80         90        100
GFQNRRLKKS QPRTWAAHTT ACPPSFLPKR KNPAYLKSVS LQEPRSRWQE
       110        120        130        140        150
SSEKRPGFRR QASLSQSIRK GAAQWFGVSG DWEGQRQQWQ RRSLHHCSMR
       160        170        180        190        200
YGRLKASCQR DLELPSQEAP SFQGTESPKP CKMPKIVDPL ARGRAFRHPE
       210        220        230        240        250
EMDRPHAPHP PLTPGVLSLT SFTSVRSGYS HLPRRKRMSV AHMSLQAAAA
       260        270        280        290        300
LLKGRSVLDA TGQRCRVVKR SFAFPSFLEE DVVDGADTFD SSFFSKEEMS
       310        320        330        340        350
SMPDDVFESP PLSASYFRGI PHSASPVSPD GVQIPLKEYG RAPVPGPRRG
       360        370        380        390        400
KRIASKVKHF AFDRKKRHYG LGVVGNWLNR SYRRSISSTV QRQLESFDSH
       410        420        430        440        450
RPYFTYWLTF VHVIITLLVI CTYGIAPVGF AQHVTTQLVL RNKGVYESVK
       460        470        480        490        500
YIQQENFWVG PSSIDLIHLG AKFSPCIRKD GQIEQLVLRE RDLERDSGCC
       510        520        530        540        550
VQNDHSGCIQ TQRKDCSETL ATFVKWQDDT GPPMDKSDLG QKRTSGAVCH
       560        570        580        590        600
QDPRTCEEPA SSGAHIWPDD ITKWPICTEQ ARSNHTGFLH MDCEIKGRPC
       610        620        630        640        650
CIGTKGSCEI TTREYCEFMH GYFHEEATLC SQVHCLDKVC GLLPFLNPEV
       660        670        680        690        700
PDQFYRLWLS LFLHAGVVHC LVSVVFQMTI LRDLEKLAGW HRIAIIFILS
       710        720        730        740        750
GITGNLASAI FLPYRAEVGP AGSQFGLLAC LFVELFQSWP LLERPWKAFL
       760        770        780        790        800
NLSAIVLFLF ICGLLPWIDN IAHIFGFLSG LLLAFAFLPY ITFGTSDKYR
       810        820        830        840        850
KRALILVSLL AFAGLFAALV LWLYIYPINW PWIEHLTCFP FTSRFCEKYE

LDQVLH
```

B

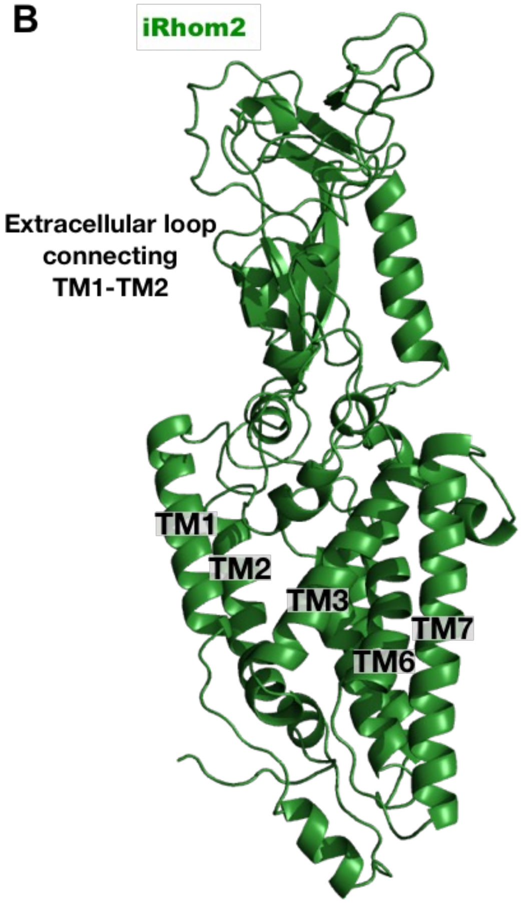

Supplementary Figure S6. Sequence (**A**) and predicted structure (**B**) of the human iR2 protein utilized in this study. (**A**) The cytoplasmic part of the protein sequence excluded in the 3D structure is shown in gray.

A

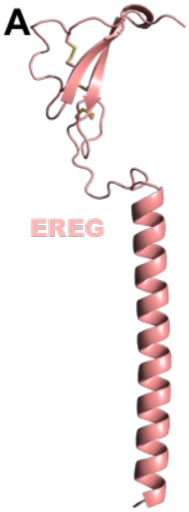

UniProtKB - O14944 (EREG_HUMAN)

10 20 30 40 50
MTAGRRMEML CAGRVPALLL CLGFHLLQAV LSTTVIPSCI PGESSDNCTA
60 70 80 90 100
LVQTEDNPRV **AQVSITKCSS DMNGYCLHGQ CIYLVDMSQN YCRCEVGYTG**
110 120 130 140 150
VRCEHFFLTV HQPLSKEYVA LTVILIILFL ITVVGSTYYF CRWYRNRKSK
160
EPKKEYERVT SGDPELPQV

B

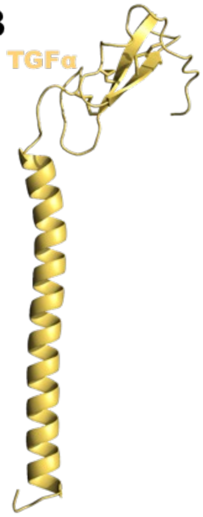

UniProtKB - P01135 (TGFA_HUMAN)

10 20 30 40 50
MVPSAGQLAL FALGIVLAAC QALENSTSPL SADPPVAAA**V VSHFNDCPDS**
60 70 80 90 100
HTQFCFHGTC RFLVQEDKPA CVCHSGYVGA RCEHADLLAV VAASQKKQAI
110 120 130 140 150
TALVVVSIVA LAVLIITCVL IHCCQVRKHC EWCRALICRH EKPSALLKGR
160
TACCHSETVV

Supplementary Figure S7. Sequence and predicted structure of human EREG (epiregulin, **A**) or human TGFα (**B**), in both cases with the part of the protein sequence excluded in the 3D structure shown in gray.

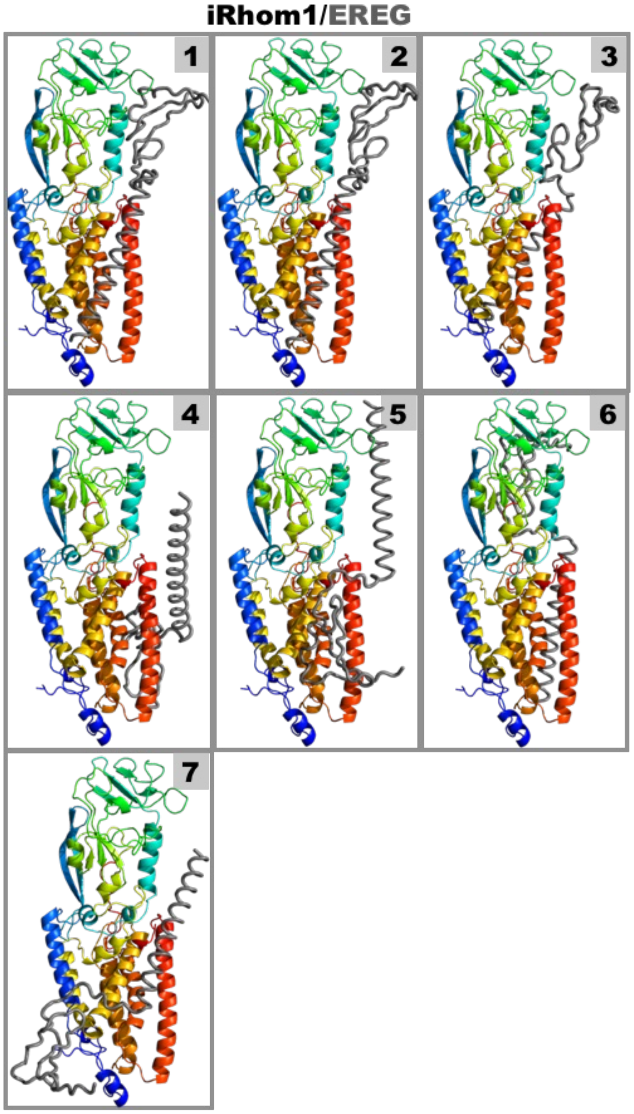

Supplementary Figure S8. Results from the docking calculation of the iRhom1/EREG complex. Molecular poses of the EREG substrate (dark gray) in the structure of the iR1 protein (rainbow color gradient). The nine poses are sorted by the values of the Autodock Vina scoring function. Poses 1 and 2 place the TMD domain of EREG in proximity of the TMD7 of iR1. Poses 3 and 6 place the TMD domain of EREG in the vicinity of TMD5. Poses 4, 5 and 7 positioned the structure of the EREG ligand in an inverted orientation with the N-terminus located at the intracellular side while the C-terminus was positioned at the extracellular side. Additionally, the EGF-domain of the ligand was located in the TMD region of the iRhom2 protein.

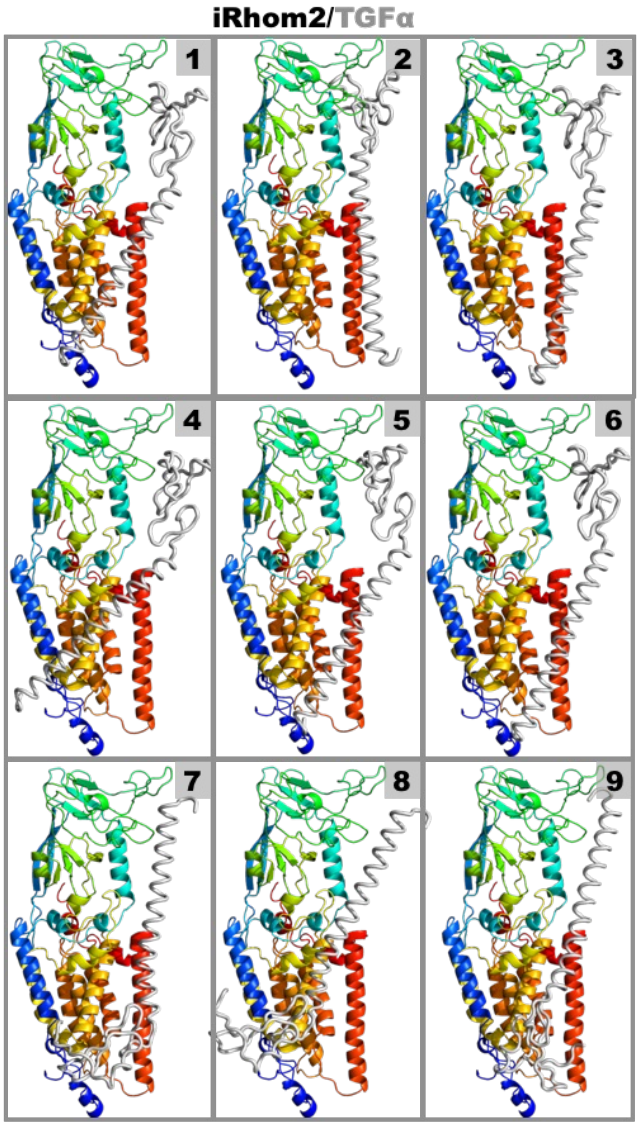

Supplementary Figure S9. Results from the docking calculation of the iRhom2/TGFα complex. Molecular poses of the TGFα substrate (light gray) in the structure of the iRhom2 protein (rainbow color gradient). The nine poses are sorted by the values of the Autodock Vina scoring function. Poses 1, 3, 5, 6 are very similar and placed the TMD domain of the TGFα ligand in the proximity of TMD7 of iR2. Poses 2 and 4 also placed the TMD domain in the vicinity of TMD7 but with an inclination of the TMD domain. The last three poses, 7, 8, and 9, position the structure of TGFα in an inverted orientation with the N-terminus located at the intracellular side while the C-terminus at the extracellular side. Additionally, the soluble extracellular domain of the ligand is located in the TM region of the iR2 protein.

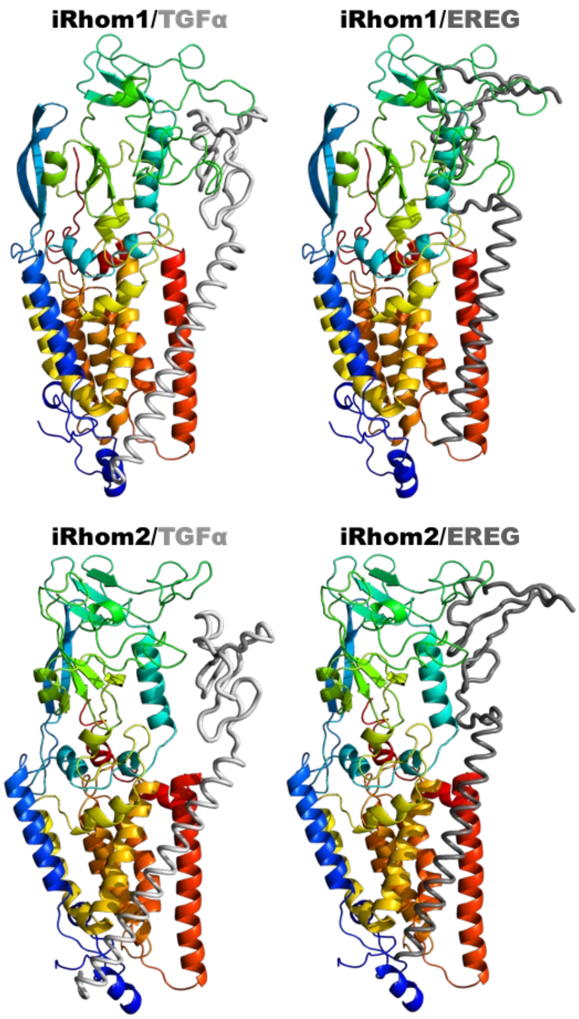

Supplementary Figure S10. Selected structures from the docking calculations of the protein complexes included in this study. The selected molecular poses for the iR1/TGFα, iR1/EREG, iR2/TGFα and iR2/EREG protein complexes are shown. As observed, the position of the TMD domains of both ligands were located in the vicinity of TMD7 in iR1 and iR2. Based on our experimental results, we favored these positions since chimeric constructs and point mutations strongly suggested the relevance of the TMD7 in modulating the substrate selectivity of iR1/2.

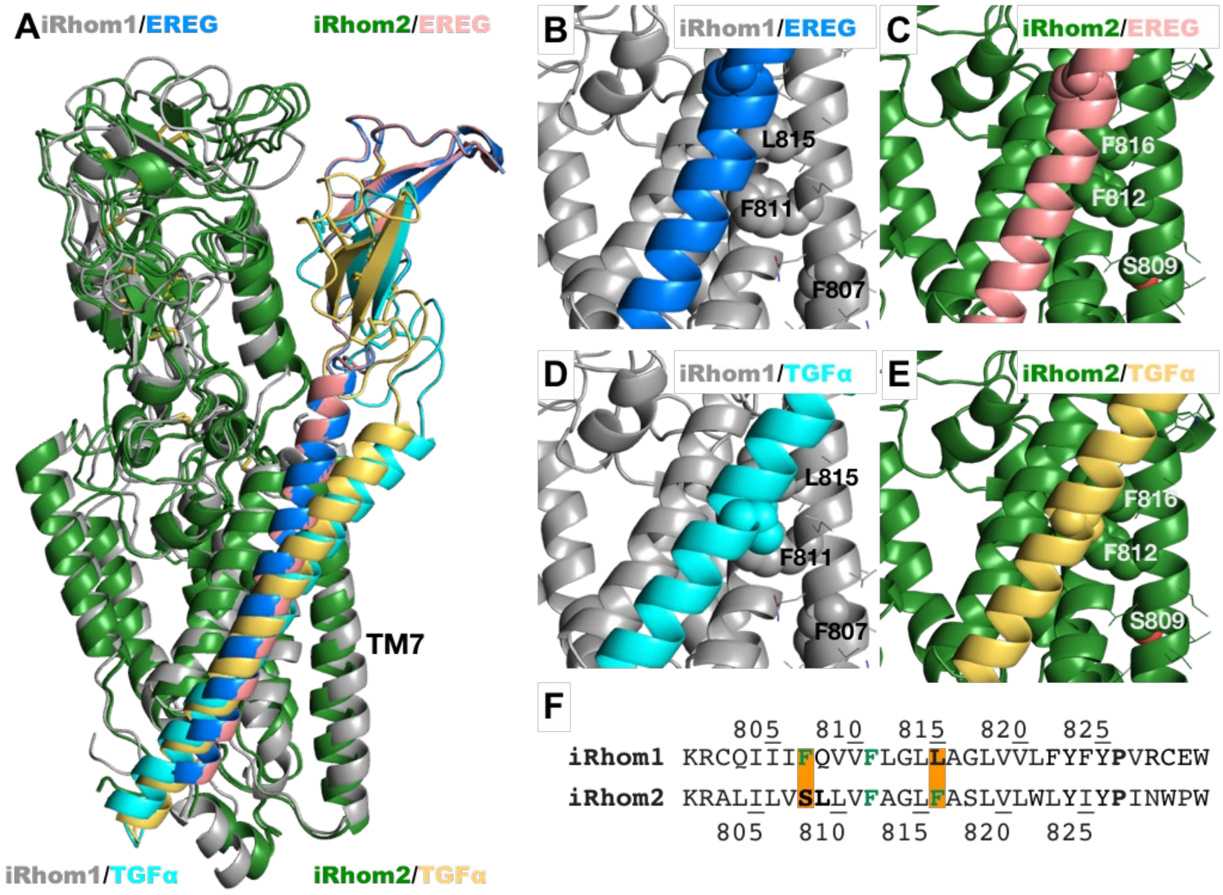

Supplementary Figure S11. (**A**) Superposition of the initial protein complexes, iR1/EREG, iR2/EREG, iR1/TGFα, and iR2/TGFα, that were investigated by unbiased MD simulations; the position of the TMD7 (TM7) of both iR1 and iR2 is indicated. Close-up representation of the possible interaction of the TMD domain of the substrates and TMD7 of iR1/EREG (**B**), iR2/EREG (**C**), iR1/TGFα (**D**), and iR2/TGFα (**E**). Residue sequences of the helix TMD7 from iR1 and iR2 (**F**), with the two functionally relevant amino acid exchanges highlighted in orange and bold letters, with Phenylalanines in green letters.

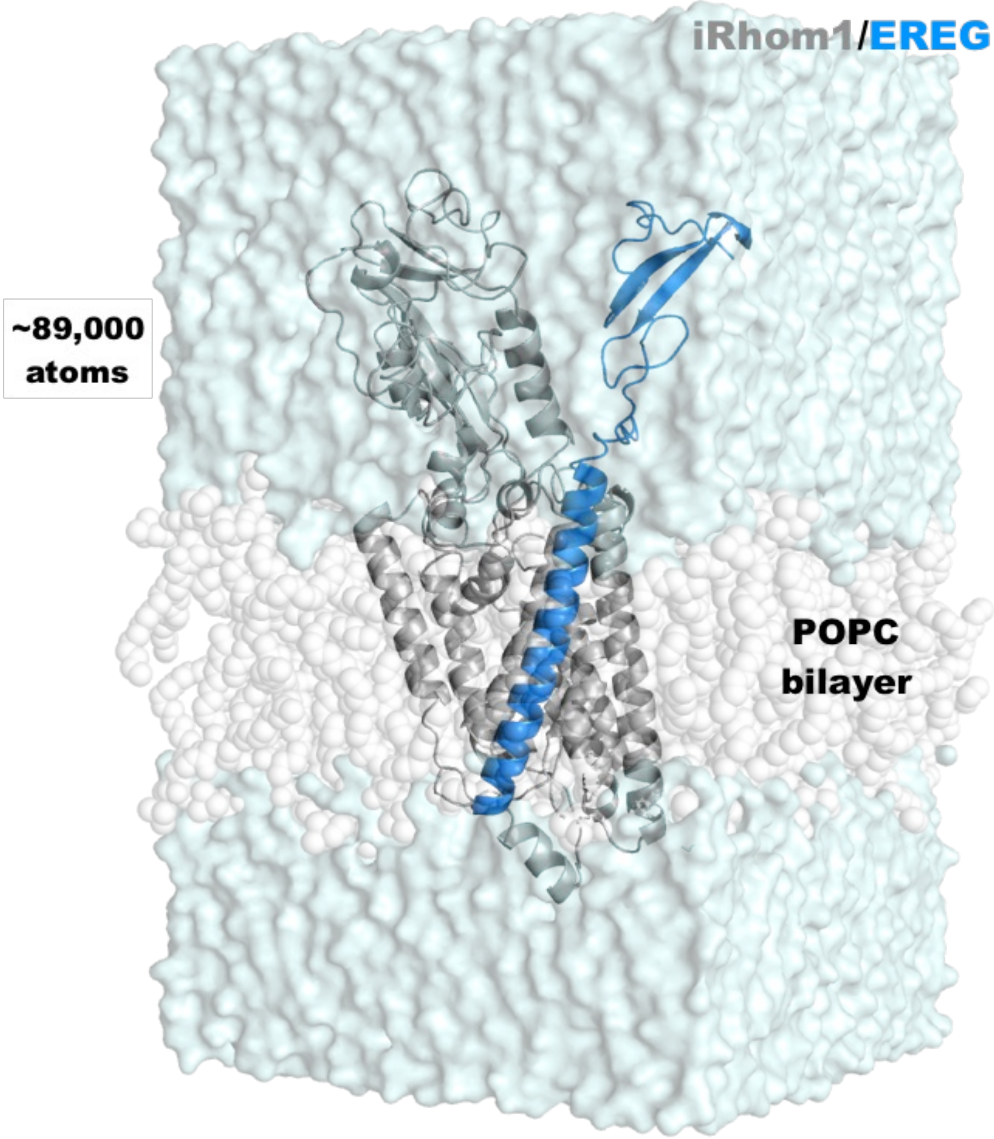

Supplementary Figure S12. Depiction of the initial iR1/EREG complex systems embedded in a hydrated POPC bilayer. The size of the systems is close to 89,000 atoms and it was investigated using unbiased all-atom MD simulations. Similar atomistic systems of the iR2/EREG, iR1/TGFα, and iR2/TGFα complexes were constructed and studied via MD simulations.

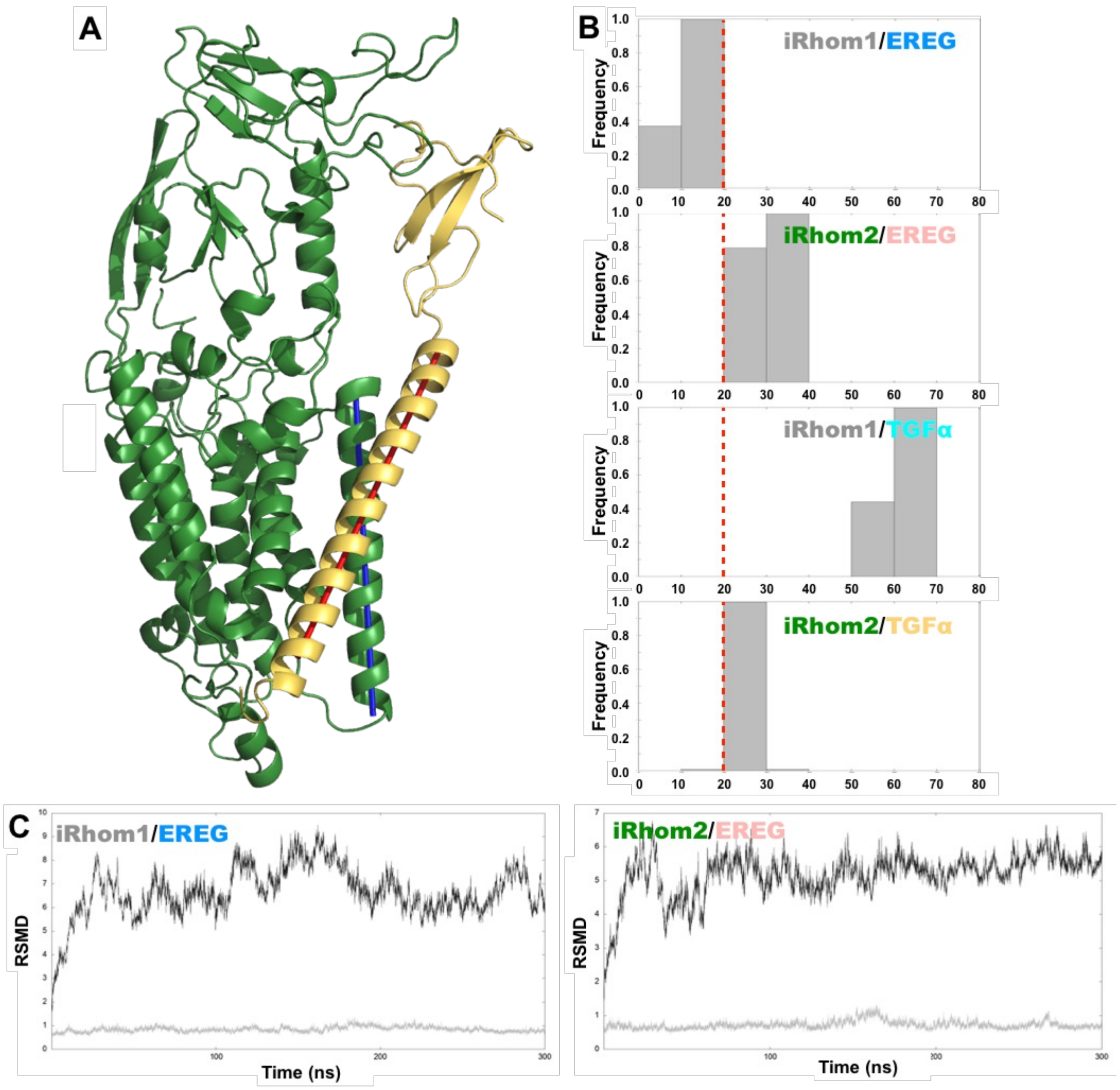

Supplementary Figure S13. Structural analysis of the iRhom/TGFα or iRhom/EREG protein complexes. (**A**) Two vectors were defining to account for the inclination of the TMD helices TMD7 (iR's) and TMD (ligands). The vectors are indicated by a blue (iR-TMD7) and a red line (EGFR-ligand TMD) in the two helices. (**B**) The inclination values in the iR1/EREG protein complex are the closest to 0° (parallel helices) while the other protein complexes explore larger values (> 20°). In the generation of the angle distributions, only the last 100ns of the simulations are considered. (**C**) The root-mean-square deviation (RMSD) for the Cα atoms in the seven TMD helices of the iRhoms (grey) as well as that for the Cα atoms in all the protein system (black) are shown (the iR1/EREG and iR2/EREG protein complexes are shown).

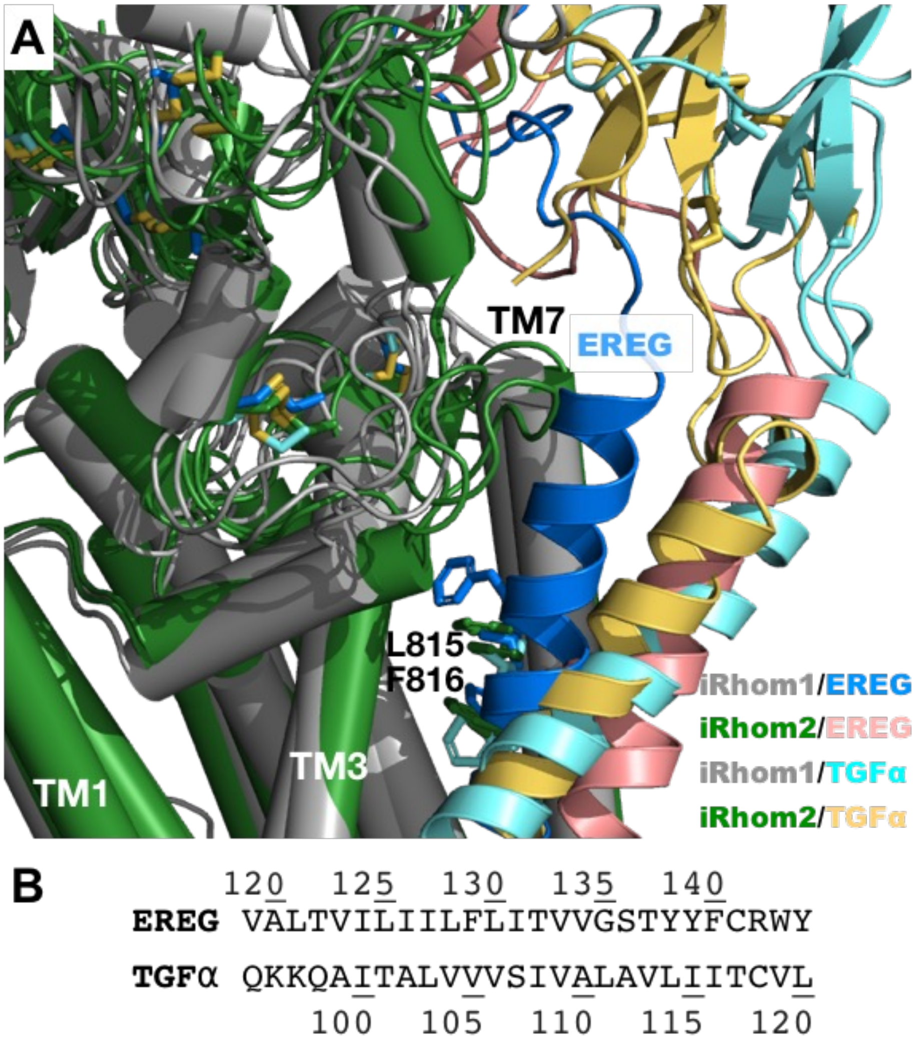

Supplementary Figure S14. (**A**) Final superimposed structures of the four protein complexes where a difference in tilt of the TMD domain of the ligands is evident. That is, while the iR2/EREG, iR1/TGFα and iR2/TGFα complexes seem to adopt a similar orientation of their TMD domain relative to the structure of iR1/2, the iR1/EREG complex exhibits significant variation. (**B**) Residue sequences of the transmembrane domains of EREG and TGFα.

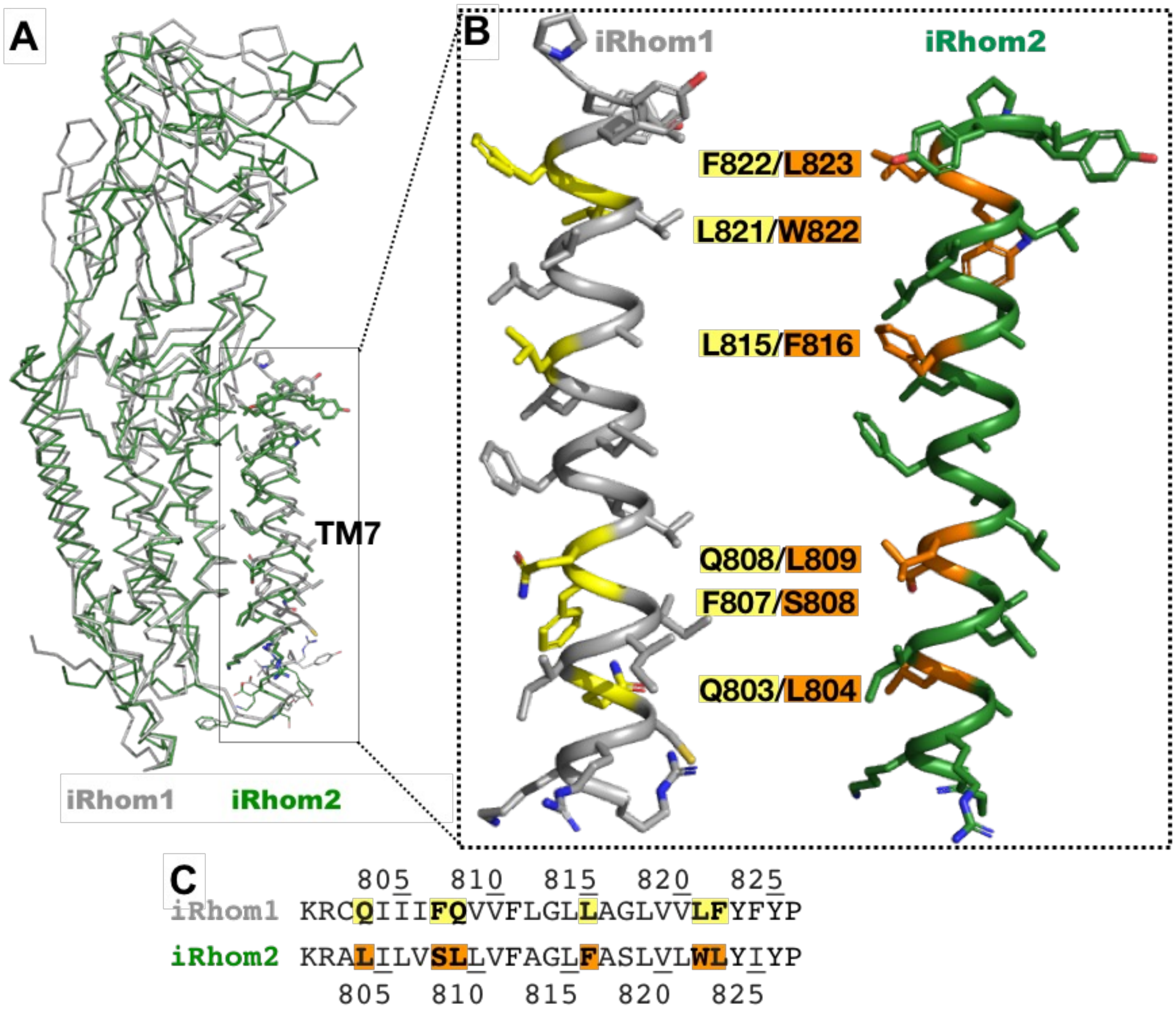

Supplementary Figure S15. Superimposed structures of iR1 and iR2 and comparison of the TMD7 in iR1 and iR2. (**A**) Superimposed structure of the seven TMDs of iR1 and iR2 with special emphasis on TMD7. (**B**) A zoom in into the structures of helix TMD7 where the main differences between the residue identity of iR1 and iR2 are highlighted in yellow and orange, respectively. (**C**) A sequence alignment of TMD7 of iR1 and iR2 where the positions that show a more significant difference in the residue identity are highlighted in yellow (iR1) and orange (iR2) (see also panel **B**).

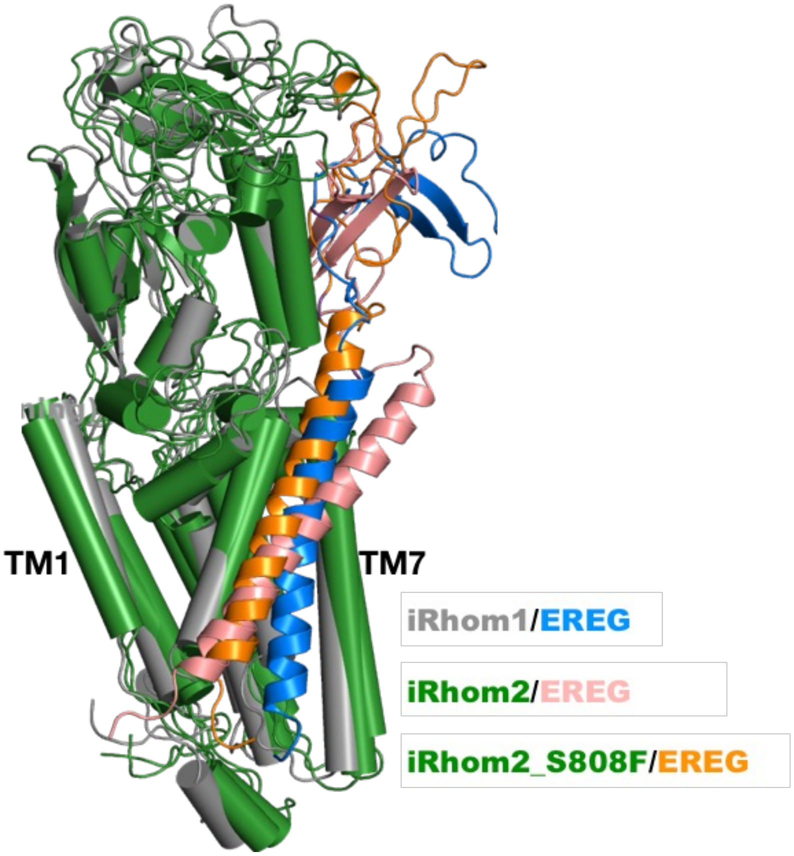

Supplementary Figure S16. Superimposed structures of the iR1/EREG, iR2/EREG and iR2-S808F/EREG protein complexes. Superimposed structures of the final stages of the MD simulations of the protein complexes, iR1/EREG, iR2/EREG and iR2-S808F/EREG, where the tilt of the TMD domain of EREG in iR2-S808F/EREG is more similar to that in iR1/EREG than in iR2/EREG. The reduction of stimulated EREG shedding by the iR2-S808F mutant could thus conceivably be caused by changes in the inclination of the EREG TMD compared to the predicted iR2/EREG complex.

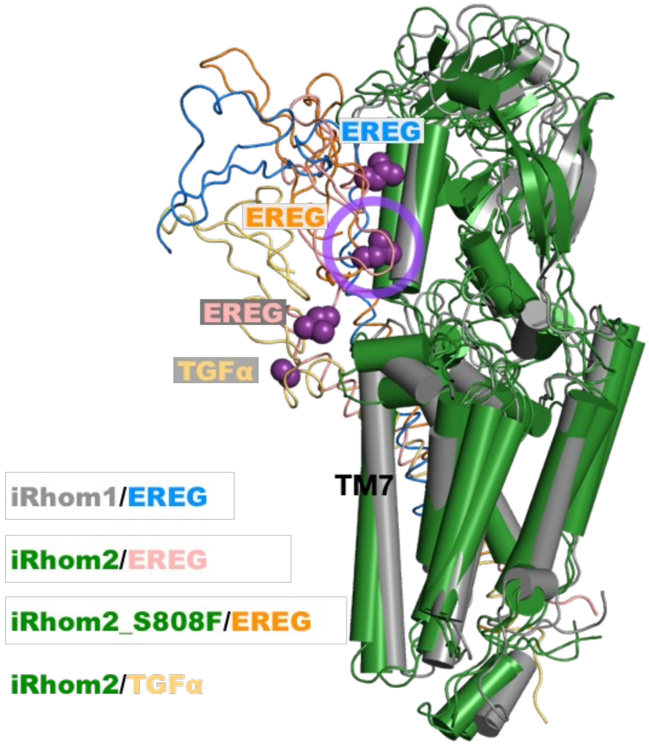

**TGFα**	-VSHFNDCPDSHTQFCFHGTCRFLVQEDKPACVCHSGYVGARCEHADLLAVVAASQKKQAITALV VVSIVALAVLIITCVLIHCCQVRKHC
**EREG**	AQVSITKCSSDMNGYCLHGQCIYLVDMSQNYCRCEVGYTGVRCEHFFLTVHQPLSKEYVALTVIL IILFLITVVGSTYYFCRWYRNRKSKE

Supplementary Figure S17. Superimposed structures of the protein complexes iR1/EREG, iR2/EREG, iR2-S808F/EREG, and iR2/TGFα. The locations of the cleavage sites in each of the substrates are indicated in purple in the diagram and also by red arrows above the sequences for the two substrates in the bottom panel. Not only does the introduction of the iR2-S808F point mutation cause a difference in the inclination of the TMD helix of EREG but the position of the EREG cleavage site in the iR2-S808F/EREG protein complex moves away from the equivalent position in the iR2/EREG and iR2/TGFα complexes.

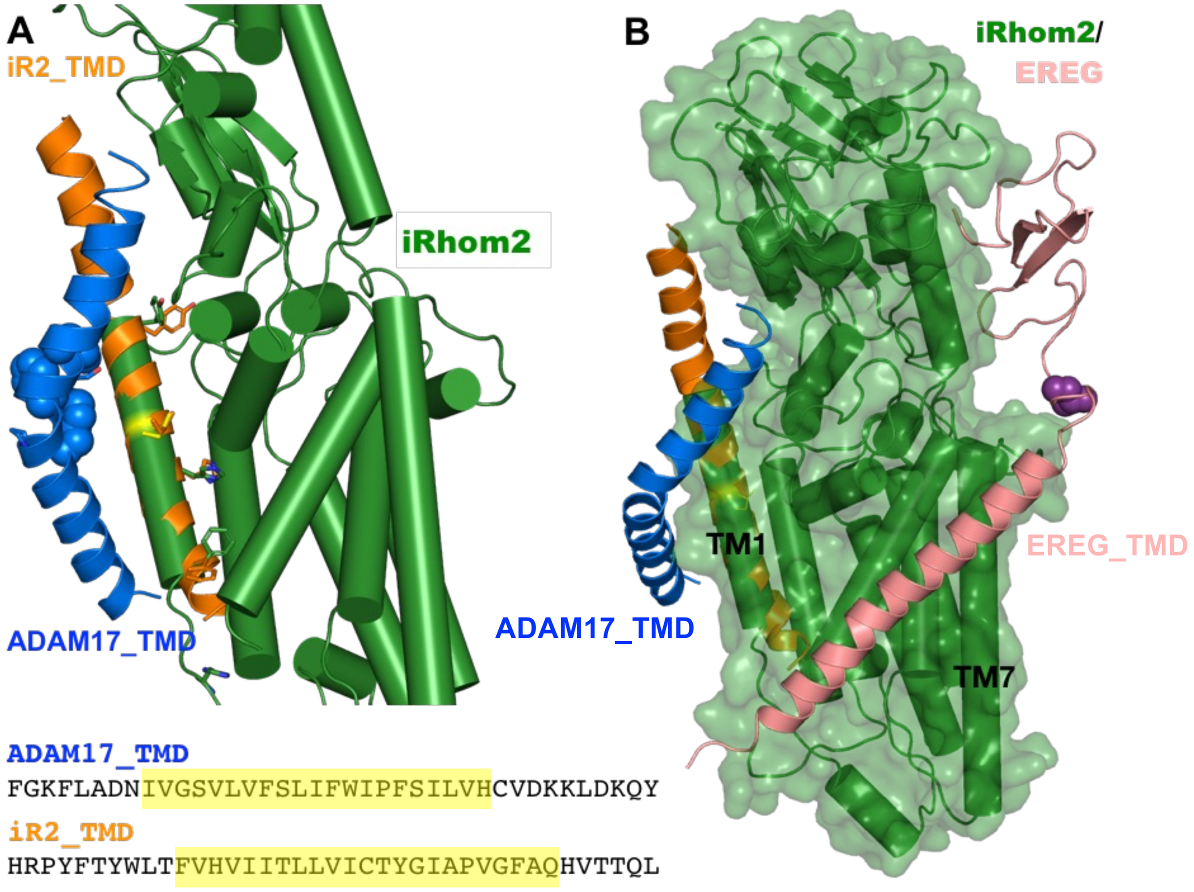

Supplementary Figure S18. Superimposed structures of the TMD domain of A17 on the current structure of the iR2/EREG complex. (**A**) Superposition of a representative structure of iR2 (green) and the previously modeled structures of the transmembrane domain of ADAM17 (blue) and the first transmembrane helix of iR2 (orange) as presented in Figure 5 of Li, et al., *Journal of Cell Science* (2017) 130:868-878. The sinecure mutation of iR2 is indicated in yellow in the predicted TMD1 structure, and the sequence of the ADAM17 TMD and the iR2 TMD1 are shown in the lower panel, with the TMDs highlighted in yellow. (**B**) Superposition of the ADAM17 TMD and iRhom2 TMD1 as in (**A**) but in the presence of the EREG structure proposed in this work. Remarkably, even though the first computational model was a simplified system due to the lack of available structural information, that is, only the TMD of ADAM17 and the first transmembrane helix of iR2 were included, there is no structural overlap between the location of the TMD domains of ADAM17 and EREG. Moreover, the bending of the ADAM17 TMD may direct its extracellular domain with the catalytic domain towards the cleavage site of the substrate (indicated in purple in **B**).
